# Wavelet analysis text classification algorithm based on typical features of data samples

**DOI:** 10.1371/journal.pone.0319747

**Published:** 2025-06-02

**Authors:** Ming Gao, Mengshi Li, Zhi Ling, Jinhao Zhong, Han Ding, Qinghua Wu

**Affiliations:** 1 School of Electrical Power Engineering, South China University of Technology, Guangzhou, China; 2 School of Electrical Engineering, Guangzhou City University of Technology, Guangzhou, China; 3 Department of Development, Zhuhai Jindao Energy Technology Company, Ltd, ZhuHai, China; Federal University of Pernambuco: Universidade Federal de Pernambuco, BRAZIL

## Abstract

Currently, traditional text feature extraction methods fail to fully capture category-specific features when handling text data with existing category labels, thereby limiting classification performance. Meanwhile, text classification methods based on wavelet analysis have yet to achieve optimal performance due to the limitations of their feature extraction and analysis techniques. To address these issues, this paper proposes two novel algorithms: (1) Average Term Frequency-Document Frequency (ATF-DF), which adopts a forward-thinking approach to comprehensively extract category-specific features from labeled text samples, resulting in class feature vectors that effectively represent the text categories; (2) Average Term Frequency-Document Frequency-Wavelet Analysis (ATF-DF-WA), which transforms class feature vectors into waveforms and utilizes wavelet analysis to extract typical class feature layer waveforms and feature layer waveforms of the text to be classified. Text classification is then performed by calculating waveform similarity. Experimental results on the THUCHNews dataset demonstrate that compared to two baseline algorithms, ATF-DF improves Precision, Recall, and F1-score by 13.71%, 28.94%, and 20.74%, respectively. Furthermore, experimental results on the THUCHNews, Sogou, and CNTC datasets indicate that ATF-DF-WA outperforms four baseline algorithms, achieving an average Precision improvement of 2.80% to 80.36%, an average Recall improvement of 0.10% to 54.65%, and an average F1-score improvement of 2.62% to 60.82%. Additionally, experimental results on the THUCHNews dataset reveal that ATF-DF-WA demonstrates advantages in both classification performance and training speed compared to baseline algorithms based on pre-trained models, highlighting its promising potential for practical applications.

## 1. Introduction

A new text feature extraction algorithm is involved in this study, and based on it, a new text classification algorithm is proposed. Therefore, the research background and motivation are discussed in two corresponding sections.

### (1) Research on text feature extraction algorithms

Text feature extraction is a core task in natural language processing, and the specific methods can be broadly classified into traditional statistical methods, word embedding methods, and deep learning methods. (a) Traditional statistical methods mainly include: ① Bag of Words (BoW) [1]: The main principle is to represent a text by counting the frequency of each word within it. The primary advantage of this method is its simplicity in implementation, while its main drawback is the loss of contextual and word order information. ② Term Frequency-Inverse Document Frequency (TF-IDF) [2]: This method evaluates the importance of a word by calculating the ratio of its frequency in a document to its frequency across the entire corpus. The primary advantage is its ability to suppress the influence of common words, while the main drawback is its disregard for semantic and contextual relationships. (b) Word embedding methods mainly include: ① Word2Vec [3]: This method uses neural networks to convert words into low-dimensional vectors. The two main models are Skip-gram and Continuous Bag of Words (CBOW) [4]. The primary advantage is its ability to capture the semantic similarity of words and contextual information, while the main drawbacks are the need for large amounts of data for training and poor performance on rare words. ② Global Vectors for Word Representation (GloVe) [5]: This method generates word vectors by constructing a word co-occurrence matrix and performing matrix factorization. The primary advantage is its ability to capture global co-occurrence information, while the main drawback is the significant amount of memory required to store the co-occurrence matrix. ③ FastText [6]: This method improves the handling of rare words by considering subword units (n-grams). The primary advantage is its ability to better manage rare words and spelling errors, while the main drawback is the increased complexity of the model. ④ Bidirectional Encoder Representations from Transformers (BERT) [7]: This is a pre-trained language model based on the Transformer architecture. The primary advantage is its ability to capture deep semantic information, while the main drawbacks are the significant computational resources and large amounts of data required for pre-training. (c) Deep learning methods mainly include: ① Long Short-Term Memory (LSTM) [8] and Gated Recurrent Unit (GRU) [9]: These methods use recurrent neural networks, which are well-suited for processing sequential data. The primary advantage is their ability to capture long-term dependencies in time series, while the main drawbacks are the slow training process and the significant computational resources required. ② Transformers [10]: These models use attention mechanisms to capture long-range dependencies. The primary advantage is their excellent performance in handling long sequences of data, while the main drawbacks are the high model complexity and the significant computational and data resources required.

However, despite the fact that word embedding methods and deep learning methods have become mainstream, traditional statistical methods still have the following key advantages: ① Lower computational resource requirements. Traditional statistical methods are simple and intuitive, requiring less computational power and not involving complex parameter adjustments. ② Better real-time performance. When computational resources are limited or real-time processing is required, traditional statistical methods offer higher computational efficiency and can provide sufficiently fast response times and reasonable accuracy for certain real-time applications. ③ Providing baseline standards and feature combinations. Traditional statistical methods can serve as baseline models to evaluate the performance of more complex models, offering researchers a simple comparative standard. Additionally, traditional statistical methods can be combined with word embedding methods to enhance model performance.

It is precisely because traditional statistical methods still possess undeniable advantages and value that some researchers continue to focus on exploring methods in this field. In recent years, research efforts have primarily centered on text feature selection methods, leading to several significant achievements. The study in [11] proposed the TF-ICF algorithm, which differs from the inverse document frequency (IDF) used in TF-IDF by employing inverse category frequency to measure the importance of terms. This approach is a theoretically concise and effective supervised feature weighting algorithm. The researcher Bekir Parlak and colleagues have also conducted a series of studies on text feature selection methods. For instance, in [12], they explored three globalizing techniques—SUM, AVG, and MAX—to transform local feature weights into global feature weights. In [13], they proposed the FCWS algorithm, which integrates the joint probability distribution of features and categories, effectively reducing the bias associated with traditional methods when handling imbalanced datasets. Additionally, in [14], they introduced the EFS algorithm, which calculates term specificity by combining category probability and corpus probability.

However, the aforementioned studies, particularly frequency-based statistical algorithms represented by TF-IDF and TF-ICF, have not fully leveraged labeled text datasets (data samples) nor comprehensively explored text classification from the perspective of category-specific feature statistics.In contrast, the FCWS algorithm proposed in [12–14] requires considering the joint probability distribution of term features and categories during the computation process, incorporating both intra-class and inter-class feature information. Similarly, the EFS algorithm takes into account both intra-class and inter-class feature distributions. These methods involve relatively complex statistical computations, which increase the difficulty of understanding and implementing the algorithms.

In view of the aforementioned issues, the first research motivation of this paper is as follows: First, this study adheres to the theoretically concise and intuitive approach of frequency-based statistical algorithms. Building upon the foundations of TF-IDF and TF-ICF, the proposed method retains the advantages of frequency-based algorithms, such as their intuitiveness, simplicity, ease of understanding, and straightforward implementation, while introducing new enhancements. Second, this paper further explores two key questions related to traditional TF-IDF and TF-ICF algorithms: ① Existing research on term frequency statistics is typically confined to individual texts. Can the scope of such statistics be expanded to labeled text datasets? In other words, can terms that frequently appear in a specific category of text better represent that category? ② Influenced by mainstream IDF-based approaches, existing studies predominantly adopt a “reverse thinking” paradigm when calculating document frequency or category frequency. Could a “positive thinking” approach be considered instead? Specifically, if a term appears more frequently within a certain category of text, does it imply a stronger representativeness of that term for the given category?

In order to answer the above two questions, the first research goal of this paper is: still focus the attention on the frequency statistical algorithm, put forward a full use of the existing category label data samples, the word frequency statistics to a certain text set, and text frequency statistics from “reverse thinking” to “positive thinking” text category feature extraction algorithm: Average Term frequency-Document frequency (ATF-DF).

Further, corresponding to the first research objectives, the supplementary discussion of the first research motivation is as follows: Currently, in the field of text feature extraction and text classification, there are already numerous labeled text datasets available. The data conditions for conducting scientific research have significantly changed compared to when TF-IDF was first introduced. Based on these advancements, supervised text feature weighting algorithms have since been developed.However, under the statistical thinking of big data, there is still room for further research on the statistical objects and angles of the existing algorithms.Therefore, this paper proposes the ATF-DF algorithm, aiming to achieve better results in extracting category-specific text features. Furthermore, this algorithm can serve as a foundation for developing new text classification algorithms.

### (2) Research on text classification algorithms

Since this paper also involves a new text classification algorithm, it is necessary to introduce the current state of research on related text classification algorithms and the motivation behind this research.

Text classification algorithms can currently be divided into two main categories: deep learning and shallow learning.

Deep learning algorithms [15] are typically implemented using convolutional neural networks (CNN), recurrent neural networks (RNN), and their variants such as LSTM and BERT. In recent years, research in this area has been highly active due to the advantages of deep learning algorithms in automatic feature extraction, handling large-scale data, managing complex nonlinear relationships, transfer learning, and pre-training mechanisms, and has become the mainstream approach in modern text classification methods. [16] provides an in-depth exploration of over 150 deep learning models and more than 40 datasets, offering a thorough study of the technical characteristics, performance, and application scenarios of various models. [17] proposes an improved Elastic Deep Autoencoder (EDA-TEC), which can simultaneously learn the manifold representation of data and clustering labels, thereby enhancing the effectiveness of clustering. [18] introduced the LyEmoBERT model, which is designed for classifying emotions in song lyrics. The study in [19] introduced the BART classifier based on the pre-trained BERT model. This classifier, which leverages a denoising autoencoder built upon the Transformer architecture, has demonstrated strong performance in classification tasks. The study in [20] indicated that the pre-trained GPT model, through fine-tuning, can also achieve outstanding performance across multiple text classification tasks.The main advantages of deep learning algorithms are: they typically outperform shallow learning algorithms in terms of classification performance when dealing with large-scale datasets and complex text classification tasks. The main drawbacks are: high data and computational resource requirements, poor interpretability, and complex hyperparameter tuning.

However, despite the significant achievements of modern text classification methods represented by deep learning algorithms, there remain several noteworthy limitations.① Deep learning algorithms typically rely on large volumes of labeled data to train complex model architectures, which limits their applicability in scenarios where data is scarce or annotation is costly. However, it is important to note that with technological advancements, fine-tuning pre-trained models such as BERT has enabled certain deep learning models to achieve significant performance even on relatively small datasets, thereby alleviating some of these limitations to a certain extent. ② Deep learning models impose substantial demands on computational resources, relying heavily on high-performance GPUs or TPUs. This presents a significant challenge for resource-constrained users, making it difficult to afford or deploy such models, particularly on widely used mobile smart devices where computational capacity is often limited. ③ The “black-box” nature of deep learning models makes their decision-making processes difficult to interpret, posing a significant drawback in applications that require clear and transparent classification criteria, such as legal or medical text classification. ④ The selection and tuning of hyperparameters in some deep learning models require extensive experimentation and domain expertise, further increasing the difficulty and cost of deployment and use.

In comparison, shallow learning algorithms often exhibit inferior performance and scalability when handling large-scale datasets or high-dimensional data compared to deep learning algorithms. However, shallow learning algorithms still have advantages in the following scenarios: ① Limited computational resources. ② Small-scale dataset scenarios. ③ High interpretability requirements. ④ Understanding data structure and patterns and facilitating feature engineering. ⑤ Providing new baseline models. ⑥ Scenarios requiring rapid development of usable classifiers.

Because of the continuing research value of shallow learning, many scholars are still committed to studying shallow learning algorithms. From a technical principle perspective, shallow learning algorithms can be categorized into methods based on mathematical statistics, decision trees, geometric approaches, and optimization, among others. Notable research outcomes include:

In the area of mathematical statistics-based methods, [21] proposes an effective K-Nearest Neighbor (KNN) classification model that uses KTree to select the optimal K value for each test sample. This method outperforms competing approaches in terms of classification precision and computational cost. [22] introduces a parallel Naive Bayes algorithm (PNBA) based on the Spark platform, which is used for large-scale Chinese text classification. In the area of decision tree-based algorithms, [23] presents a decision tree smoothing algorithm that enhances decision tree performance, stabilizes probability estimates, converts the model into easily interpretable rule sets, and is suitable for large-scale datasets. In the area of geometric methods, [24] found that the SVM classifier achieved an excellent F1 score above 86.26% in text classification tasks. [25] compares SVM algorithms with artificial neural network algorithms and finds that selecting an appropriate feature set is crucial for accurate classification. In the area of optimization methods, [26] proposes an improved Discrete Layered Chicken Algorithm (IDLCA), which enhances feature selection and text classification performance through adaptive operators.

In addition to the methods mentioned above, new approaches to text classification algorithms continue to evolve. One noteworthy research trend is that a small number of scholars have begun to introduce wavelet analysis tools into the field of text classification. This approach, which can be referred to as wavelet analysis-based methods, has produced some notable research outcomes, including: [27] uses wavelet analysis to reduce the dimensionality of text feature space vectors.[28] first constructs a semantic network from the text, converts it into an image using a template, and then applies wavelet analysis to extract image features for text classification. This study represents an active exploration of using wavelet analysis tools for text classification.

However, existing research still faces the following issues: The approach in [27] focuses solely on using wavelet analysis for dimensionality reduction, rather than directly performing text classification calculations. [28] requires converting the text into an image before applying wavelet analysis for classification, resulting in a more complex algorithmic logic. Consequently, the final text classification precision is only 15.404%, which is even lower than that of earlier classification algorithms. Overall, current research on wavelet analysis in the field of text classification is limited and has not yet achieved the desired results.

Therefore, the second research objective of this paper is to develop a new text classification algorithm, Average Term Frequency-Document Frequency-Wavelet Analysis (ATF-DF-WA), based on the ATF-DF text feature extraction algorithm proposed in this study.

The second research motivation of this paper can be described as follows. From an informatics perspective, both text and waveforms can be regarded as forms of encoding that can be mutually transformed, allowing the integration of analytical theories and tools from their respective domains. Given the widespread application and remarkable effectiveness of wavelet analysis in scientific research, this paper develops a keen interest in applying wavelet analysis to text classification. By thoroughly analyzing the limitations of existing studies, this research aims to advance improvements in this field. Specifically, the technical motivation is to extract category-specific features from text using the ATF-DF algorithm, represent these features as vectors, and subsequently transform them into waveforms for further analysis. Wavelet analysis is then employed to analyze these waveforms to accomplish the text classification task.

Therefore, the ATF-DF-WA algorithm is divided into two stages. The first stage is the feature extraction phase, where the ATF-DF algorithm is used to fully extract features from large-scale, multi-class text samples with existing category labels, resulting in class-typical feature vectors. The second stage is the text classification phase. In this stage, feature vectors for the samples to be classified are generated based on the class-typical feature vectors.These feature vectors undergo wavelet analysis (WA) to produce feature layer waveforms, and text classification is completed by calculating the similarity between these waveforms and the class-typical feature layer waveforms.

The proposed ATF-DF-WA algorithm, compared to shallow learning algorithms, not only inherits the efficient computational characteristics of traditional statistical methods but also enhances the accuracy of feature extraction by incorporating category label information. Moreover, by applying wavelet analysis theory to text classification, the algorithm further improves classification performance through waveform similarity calculations.Compared to deep learning algorithms, the ATF-DF-WA algorithm does not require extensive computational resources or large-scale training data, making it suitable for deployment on devices with limited computational power. Additionally, it offers higher interpretability and real-time responsiveness. Its specific advantages include: ① High computational efficiency, making it suitable for resource-constrained devices, particularly excelling in low-resource environments such as mobile smart terminals. ② Strong interpretability, which makes it well-suited for tasks that require clear and transparent classification criteria. ③ No requirement for large-scale training data and high-performance hardware, making it an ideal solution for data-scarce and low-cost application scenarios. ④ Good real-time performance, enabling fast responses, which is beneficial for real-time applications.

Therefore, ATF-DF-WA provides an effective alternative solution to both deep learning and traditional shallow learning algorithms for application scenarios that demand high efficiency, low resource consumption, and strong interpretability. This method demonstrates significant potential for practical applications, particularly in various text classification tasks involving labeled text datasets. For instance, in domains such as news, law, and medicine, text category features often exhibit certain regularities. By constructing category feature vectors and integrating wavelet analysis, ATF-DF-WA can accurately capture these features, achieving a significant improvement in classification performance while maintaining low computational cost.

The main contributions of this paper are threefold

① It introduces the ATF-DF algorithm, a traditional statistical method for feature extraction. It is designed for large-scale data sample scenarios, focusing on extracting category features from labeled data samples. Compared to other traditional statistical methods, this algorithm provides more accurate extraction of text category features.
② The proposed ATF-DF-WA algorithm is a wavelet analysis-based method in text classification. In the first stage, it fully extracts text category features using the ATF-DF algorithm. In the second stage, it transforms the text classification problem into a waveform analysis problem, utilizing wavelet analysis tools for text feature extraction and analysis. This approach effectively leverages the strengths of wavelet analysis in signal feature processing and analysis. As a result, the evaluation metrics for text classification using this algorithm show significant improvements compared to traditional text classification algorithms.
③ The proposed ATF-DF-WA algorithm, compared to deep learning-based text classification algorithms, not only fully utilizes large datasets but also maintains the advantages of interpretability and understandability in the classification process, avoiding the black-box issue. Additionally, the algorithm requires significantly less training data and computational resources compared to deep learning algorithms. While achieving significant improvements in classification performance, it also retains the benefits of shallow learning algorithms.

The remainder of this paper is organized as follows: Chapter 1 introduces the theoretical background related to this study; Chapter 2 presents the theory and experimental results of the ATF-DF algorithm; Chapter 3 discusses the theory and experimental results of the ATF-DF-WA algorithm; and Chapter 4 provides a summary of the conclusions, identifies the limitations of the study, and outlines directions for future research.

## 2 Background theoretical

### 2.1 TF-IDF feature extraction algorithm

In text classification tasks, the first stage is typically feature extraction. Commonly used text feature extraction algorithms include TF-IDF, N-gram models, Word2Vec, and $X_{2}$ -statistics. Among these, the TF-IDF algorithm is particularly representative and is the primary focus of comparative research in text feature extraction in this paper.

The TF-IDF feature extraction algorithm combines two factors: term frequency (TF) and inverse document frequency (IDF) to calculate the weight of each word. The calculation equation is as follows:


$$w_{kj}=tf_{kj}\cdot \log\left(\frac{\left|D\right|}{1+\left|d_{k}\right|}\right)$$ (1)

Where $w_{kj}$ represents the TF-IDF weight of the *k* -th term $t_{k}$ in the *j* -th document, $tf_{kj}$ denotes the frequency of $t_{k}$ in the *j* -th document, $\left|D\right|$ represents the total number of documents in the corpus, and $\left|d_{k}\right|$ is the number of documents in $\left|D\right|$ that contain $t_{k}$.

### 2.2 Term frequency-inverse category frequency(TF-ICF)feature extraction algorithm

Compared to the TF-IDF algorithm, TF-ICF [11] places greater emphasis on the distribution of terms at the category level rather than across the entire document collection. The core idea of TF-ICF is that terms appearing in fewer categories are more distinctive for text classification and should therefore be assigned higher weights. The equation is shown in (2).


tf⋅icf(t,d)=tf(t,d)×icf(t) (2)

Where, tf(t,d) represents the frequency of term *t* in document *d*, while icf(t) is the inverse category frequency of term *t*, calculated using the following equation:


$$icf(t)=\log(\frac{\left|c\right|}{cf(t)})$$ (3)

Where $\left|c\right|$ represents the total number of categories in the training corpus, and cf(t) denotes the number of categories in which the term *t* appears.

### 2.3 Wavelet analysis

Wavelet Analysis [29] can decompose a signal into sub-signals of different frequencies, allowing for a better understanding and analysis of the signal’s characteristics and properties. The principle involves using a set of functions called wavelet bases, where the original signal is convolved with the wavelet bases to obtain a set of wavelet coefficients. By adjusting and processing these coefficients, the signal can be decomposed and reconstructed. Wavelet bases are a specific set of function families, with common examples including Morlet wavelets, Haar wavelets, and Daubechies wavelets. By selecting different wavelet bases and adjusting the number of decomposition levels, wavelet analysis can be better tailored to different types of signal processing tasks. Continuous Wavelet Transform (CWT) is used to decompose a signal into wavelet components at different scales and frequencies. By convolving the signal with a wavelet basis function, CWT generates wavelet coefficients under varying scales and translations, thereby characterizing the energy distribution of the signal in both the frequency and time domains. The equation is as shown in (4):


$$W\left(a,b\right)=\int \left[f\left(t\right)\psi^{*}\left[\frac{t-b}{a}\right]\right]dt$$ (4)

where $W\left(a,b\right)$ represents the wavelet coefficient at scale parameter *a* and translation parameter *b*, $f\left(t\right)$ is the original signal, and $\psi^{*}$ denotes the conjugate function of the wavelet basis.

### 2.4 Pearson correlation coefficient

The Pearson correlation coefficient between two variables *x* and *y* is denoted as *r* or $r_{xy}$, and its equation is shown in (5):


$$r_{xy}=\frac{\mathrm{cov}(x,y)}{\sqrt{\mathrm{var}(x)}\cdot \sqrt{\mathrm{var}(\text{y})}}$$ (5)

where cov(x,y) is the covariance between x and *y*, and var(x) and var(y) are the variances of x and *y*, respectively.

The Pearson correlation coefficient ranges from [−1, 1]. Its sign indicates the direction of the relationship between the variables, while the magnitude (closer to −1 or 1) reflects the strength of this relationship. A value of −1 indicates a perfect negative linear relationship, 0 signifies no linear relationship, and 1 denotes a perfect positive linear relationship. In academic research, this coefficient is often used to assess the similarity between two vectors or waveforms.

## 3 Average term frequency-document frequency (ATF-DF) algorithm

### 3.1 Algorithm concept

Assuming that in the training set, there are *M* classes of text with known category labels, the general process of the ATF-DF algorithm is as follows:

First, all texts with known categories in the training set are tokenized into words. After tokenization and preprocessing, *M* class-specific lexicons (hereinafter referred to as lexicons) are constructed.

Next, the ATF-DF values for all terms are calculated from the lexicons, resulting in *M* class-specific feature vectors.

Finally, based on the calculated results, the terms are sorted in descending order according to their ATF-DF values, resulting in *M* class-specific feature vectors arranged in descending order.

**Fig 1:**
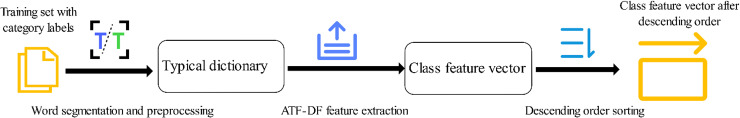
Schematic Diagram of the ATF-DF Algorithm Concept.

**Fig 2:**
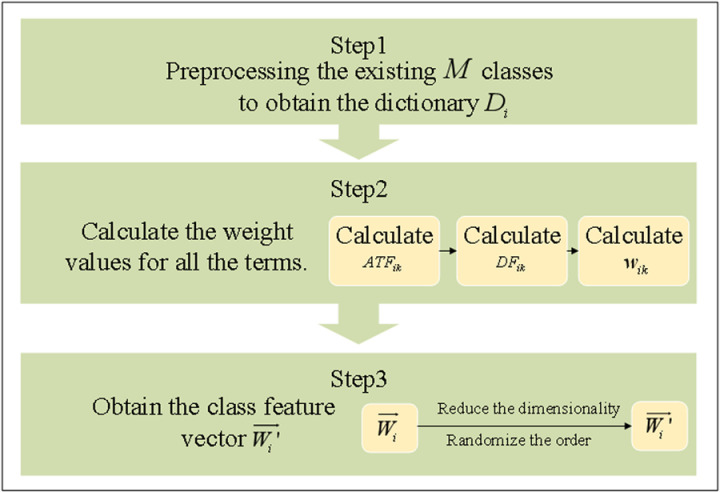
ATF-DF Algorithm Framework Diagram.

The concept of the ATF-DF algorithm is illustrated in Fig 1, and the framework of the algorithm is depicted in Fig 2.

### 3.2 The theory and procedure of the ATF-DF-WA algorithm

**Fig 3:**
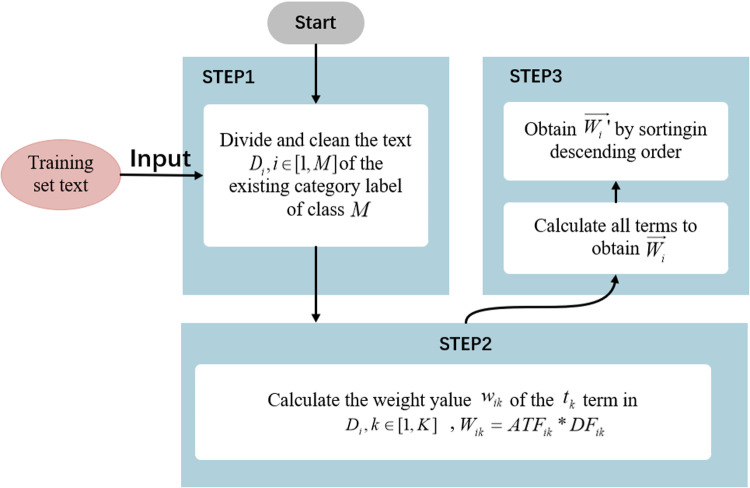
ATF-DF Algorithm Flowchart.

Expanding on Fig 2, the process of the ATF-DF algorithm is shown in Fig 3.

As shown in Fig 3, the ATF-DF algorithm consists of three main steps:

STEP 1: Assuming the text is categorized into *M* classes, after tokenization, cleaning, and other preprocessing steps, the lexicon $D_{i}$$\left(i\in \left[1,M\right]\right)$ is obtained.

STEP 2: Calculate the class-specific feature weights for all terms in $D_{i}$ and obtain the class feature vector $\vec{W_{i}}$.STEP 2 consists of two specific sub-steps.

#### (1) Calculate the class-specific feature weights $w_{\text{i}k}$.

Assume that $D_{i}$ contains a total of *K* terms, where the *k* -th term is $t_{k}$, k∈(1,K), and its class-specific feature weight $w_{\text{i}k}$ is calculated in the following order:

① Calculate ${\text{ATF}}_{ik}$, where the subscript *i* represents the corresponding *i* -th class lexicon, and *k* represents the *k* -th term in $D_{i}$. ${\text{ATF}}_{ik}$ is used to represent the class-specific feature weight of $t_{k}$ with respect to $D_{i}$ at the term level. ATF is the abbreviation for Average Term Frequency.

The traditional TF algorithm is used to extract term frequency features from individual texts and is not suitable for scenarios involving large datasets with multiple texts of known categories. The reasons are as follows: If TF is used to calculate the frequency of $t_{k}$ in $D_{i}$, it may lead to instability in frequency. That is, the frequency of $t_{k}$ in individual texts does not tend toward a stable value; it may be high in one text but low in others, making the final frequency feature difficult to determine.

The core idea of ATF is that if a term has a higher average frequency within a specific category, then that term has a stronger ability to distinguish that category, and is therefore assigned a higher weight.

Therefore, this paper proposes calculating the average term frequency (ATF) of $t_{k}$ in $D_{i}$ to represent the class-specific feature weight of $t_{k}$ with respect to $D_{i}$ at the term level. The calculation equation is as follows:


$${\text{ATF}}_{ik}=\frac{\sum_{j=1}^{J}tf_{kj}}{J}$$ (6)

Here, the subscript *i* in ATF indicates that the class-specific feature weight corresponds to the *i* -th class text $D_{i}$, *k* is the subscript for $t_{k}$, *j* represents the *j* -th text in $D_{i}$, *J* is the total number of texts in $D_{i}$ that contain $t_{k}$, and $tf_{kj}$ represents the TF value of $t_{k}$ in the *j* -th text.

② Calculate ${\text{DF}}_{ik}$, where the subscript *i* represents the corresponding *i* -th class lexicon, and *k* is the subscript for $t_{k}$. ${\text{DF}}_{ik}$ is used to represent the class-specific feature weight of $t_{k}$ with respect to $D_{i}$ at the document category level. DF is the abbreviation for Document Frequency.

The traditional IDF algorithm is based on the idea that if $t_{k}$ appears in only a few texts, it has a strong discriminative ability and can be used to identify or classify texts. It is evident that this algorithm is only applicable to scenarios where the text set does not have category labels. However, the scenario in this paper involves calculating the term frequency features of $t_{k}$ within a text set that already has category labels.

Therefore, this paper proposes calculating the ${\text{DF}}_{ik}$ value, with the core idea being: since $D_{i}$ already has category labels, the approach is the exact opposite of the traditional IDF algorithm. The more frequently $t_{k}$ appears in the texts within $D_{i}$, the stronger the ability of $t_{k}$ to represent the category features. This study uses ${\text{DF}}_{ik}$ to represent the weight of $t_{k}$ in terms of its ability to characterize the text category $D_{i}$ at the text frequency level. The calculation equation is as follows:


$${\text{DF}}_{ik}=\frac{\left|d_{ik}\right|}{\left|D_{i}\right|}$$ (7)

Here, the numerator $\left|d_{ik}\right|$ represents the total number of texts in $D_{i}$ that contain $t_{k}$, while the denominator $\left|D_{i}\right|$ is the total number of texts in $D_{i}$.

③ Calculate the ATF-DF value, denoted as $w_{ik}$, which is used to comprehensively represent the class-specific feature weight of $t_{k}$ with respect to $D_{i}$ at both the term frequency and document category levels. The calculation equation is shown in (8):

$$W_{ik}={\text{ATF}}_{ik}\cdot {\text{DF}}_{ik}$$ (8)

Here, the subscript *i* indicates that $w_{ik}$ corresponds to the *i* -th class lexicon, and *k* is the subscript for $t_{k}$.

#### (2) Calculate the class feature vector $\vec{W_{i}}$.

Assuming $D_{i}$ has *K* terms, after calculating all terms to obtain $w_{ik}$$\left(k\in \left[1,K\right]\right)$, the result is $\vec{W_{i}}$, which is referred to as the class feature vector corresponding to $D_{i}$. Its specific composition is as follows:

$\vec{W_{i}}$ contains *K* elements, each of which consists of a two-dimensional array. In the two-dimensional array, the first sub-element is the term, and the second sub-element is the class-specific feature weight corresponding to that term. The *k* -th element of $\vec{W_{i}}$ can be represented as $\left[t_{ik},w_{ik}\right]$.

STEP 3: Calculate and obtain the class feature vectors arranged in descending order.

Sort each element of $\vec{W_{i}}$ in descending order based on the value of $w_{ik}$ to obtain the class feature vector $\vec{{W^{\prime }}_{i}}$.

### 3.3 Comparison and analysis with related algorithms

In Section 3.2, while explaining the specific calculation steps of ATF-DF, two significant differences between ATF-DF and the TF-IDF algorithm have been highlighted:

(1) The method of term frequency calculation is different: TF calculates the term frequency weight of a specific term within a single text of an unknown category, whereas ATF calculates the average term frequency weight of a specific term within a text set of a known category (which includes multiple texts). The core idea is: If a certain term has a higher average frequency within that category, it has a stronger ability to represent that text category, and therefore, it is assigned a higher weight.
(2) The method of document frequency calculation is different: The basic theory behind IDF is that if a term appears frequently in many texts, it indicates that the term does not have a strong ability to distinguish between text categories. However, since DF is calculated based on a text set with known categories, its basic theory is exactly the opposite of IDF: If a certain term appears more frequently in a specific category of texts, it indicates that the term has a better ability to represent that text category.

Additionally, the ATF-DF algorithm has two significant differences from the TF-ICF described in Section 2.2:

The method of term frequency calculation is different: The differences between the TF and ATF algorithms are as previously described.

The method of category frequency calculation is different: ICF calculates the inverse category frequency, with its core idea being: The fewer times a term appears in a category, the more distinctive it is for text classification. In contrast, the calculation of the DF value has an opposing core idea: If a certain term appears more frequently in a specific category of texts, it indicates that the term has a better ability to represent that text category.

A comprehensive comparison of the TF-IDF, TF-ICF, and ATF-DF algorithms is shown in Table 1.

**Table 1:** Comparison of TF-IDF, TF-ICF, and ATF-DF Algorithms.

Algorithm Name	Algorithm Objective	Term Frequency Calculation Objective	Basic Theory of Document Frequency and Category Frequency Calculation	Feature Value Calculation
TF-IDF Algorithm	Extract feature words with discriminative power from individual documents with unknown categories.	Calculate the term frequency weight of a specific term within an individual text of an unknown category.	If a term appears frequently in many texts, its ability to distinguish between text categories is weak.	The product of TF value and IDF value
TF-ICF Algorithm	Extract feature words with discriminative power from individual documents without labeled categories.	Calculate the text category representative weight of a specific term within a text set of a known category.	If a term appears less frequently in a certain category of texts, it indicates that the term has a stronger ability to represent that text category.	The product of TF value and ICF value
ATF-DF Algorithm (Our algorithm)	Extract text category feature vectors from multiple texts that already have category labels.	Calculate the category representative weight of a specific term within a text set of a known category.	If a term appears more frequently in a certain category of texts, it indicates that the term has a stronger ability to represent that text category.	The product of ATF value and DF value

### 3.4 ATF-DF algorithm experiments

#### 3.4.1 Experimental design and selection of baseline algorithms.

***Experimental Objective:*** To validate the effectiveness of ATF-DF in the feature extraction algorithm for text categorization.

***Experimental Design:*** This study aims to assess the impact of different feature extraction methods on text classification performance. Specifically, we compare the classic TF-IDF with the ATF-DF algorithm in baseline text classification tasks.

***Selection of the Baseline Algorithm:*** Since it is necessary to compare the performance of TF-IDF and ATF-DF within the same baseline text classification algorithm, the baseline text classification algorithm must meet the following criteria: In the feature extraction phase of this algorithm, while maintaining the basic principles of the original algorithm, it must support replacing the traditional TF-IDF algorithm with the ATF-DF algorithm for experimentation.

Among the candidate baseline algorithms, this paper examines three representative shallow learning algorithms: KNN, Naive Bayes Multinomial (NBM), and SVM, and analyzes whether each of them meets the above criteria:

① KNN: Since both TF-IDF and ATF-DF generate feature vectors that can be used for distance calculation, and there are no independence assumptions required, KNN is selected as a baseline algorithm.
② NBM: Since a key step in the NBM algorithm is calculating the probability of a term appearing in a document given its known category, which aligns with the fundamental concept of the ATF-DF algorithm, ATF-DF can be used to replace the original feature extraction algorithm for experimental comparison. Therefore, NBM is selected as a baseline algorithm.
③ SVM: Since the SVM algorithm heavily relies on the independence of feature vectors, and the introduction of text category information in ATF-DF may increase the correlation between feature vectors, potentially affecting SVM’s requirement for feature independence, SVM is not selected as a baseline algorithm.

To rigorously compare the performance differences between TF-IDF and ATF-DF within the same baseline algorithm, the feature extraction process in both the KNN and NBM baseline algorithms is consistently implemented using the TF-IDF algorithm. For the sake of clarity and precision, these two baseline algorithms are henceforth referred to as TF-IDF-KNN and TF-IDF-NBM, respectively.

***Experimental Evaluation and Analysis***: By evaluating the experimental results of the baseline algorithms, the effectiveness of the ATF-DF algorithm is analyzed, leading to the formation of conclusions.

Additionally, the classification performance of the new text classification algorithm, based on ATF-DF, which is introduced later in this paper, further validates the effectiveness of ATF-DF from another perspective.

#### 3.4.2 Description of experimental datasets and environmental parameters.

The experimental dataset used in this study is the THU Chinese Text Classification (THUCHNews) [30] corpus, (Download link: http://thuctc.thunlp.org/). Developed by the Natural Language Processing Laboratory of Tsinghua University, THUCHNews is a Chinese text classification dataset containing approximately 740,000 text samples.This corpus is renowned in the field of Chinese studies for its comprehensive text categories, extensive scale, and high quality, making it a commonly used resource in academic research. The corpus encompasses a total of 14 news categories, with their respective data categories displayed in Table 2. To facilitate training and testing, 80% of the text within each category is randomly selected to form the training dataset, while the remaining 20% is designated as the test dataset.

**Table 2:** Data categories of the experimental corpus.

i (Category number)	Category	Number of text	i (Category number)	Category	Number of text
1	Sports	79102	8	Current Affairs	63086
2	Entertainment	92632	9	Constellation	3578
3	Furniture	32586	10	Games	24373
4	Lottery	7588	11	Social	50849
5	Real-Estate	20050	12	Technology	84461
6	Education	41936	13	Stock	154398
7	Fashion	13368	14	Finance	37098

The program was run on a computer with 16.0 GB of RAM, an AMD Ryzen 7 6800H with Radeon Graphics processor, and a clock speed of 3.20 GHz. The dependencies include scikit - learn (version 0.24.2) and pywt (version 1.1.1).

#### 3.4.3 Sub-experiment 1: ATF-DF algorithm text category feature extraction experiment and result analysis.

***The experimental steps are as follows:*** After segmenting all texts in the training dataset (The Chinese text segmentation tool employed is jieba [31]) and performing cleaning, a lexicon corresponding to 14 news categories, denoted as $D_{i}$$\left(i\in \left[1,14\right]\right)$ is obtained.

The key technical details of this step include:(a) The Jieba segmentation tool (version 0.42.1) is used in precise mode to perform Chinese text segmentation, ensuring the accurate extraction of each term.(b) The Chinese stopword list provided by Harbin Institute of Technology (stopword.txt) is used to remove common words with little semantic value, such as “的” (de) and “是” (shi), ensuring that meaningless terms do not interfere with the text analysis.(c) The text is cleaned by removing all non-Chinese characters, including punctuation marks, numbers, and special symbols. Additionally, meaningless whitespace characters are eliminated, and the text content is uniformly converted to lowercase, which is particularly applicable in scenarios containing English characters.(d) Texts with a length of fewer than 5 characters are filtered out, along with those containing a large number of noisy characters such as repeated punctuation marks, to ensure data quality and improve the effectiveness of subsequent text processing and analysis.

Using the ATF-DF algorithm, the term weights for all terms in $D_{i}$ are calculated to obtain $w_{ik}$, and the category feature vectors $\vec{W_{i}}$$\left(i\in \left[1,14\right]\right)$ are derived.

The term weights $\vec{W_{i}}$ are sorted in descending order to obtain the category feature vector $\vec{{W^{\prime }}_{i}}$.

Due to the high dimensionality of $\vec{{W^{\prime }}_{i}}$, for ease of presentation, the top 500 elements of all $\vec{{W^{\prime }}_{i}}$ vectors are retained, as shown in Table 3.

**Table 3:** 14 Category Feature Vectors.

$\vec{{W^{\prime }}_{i}}$	$\left(t_{i1},w_{i1}\right)$	$\left(t_{i2},w_{i2}\right)$	$\left(t_{i3},w_{i3}\right)$	$\left(t_{i4},w_{i4}\right)$	$\left(t_{i5},w_{i5}\right)$	$\left(t_{i6},w_{i6}\right)$	$\left(t_{i500},w_{i500}\right)$
$\vec{{W^{\prime }}_{1}}$	(competition, 0.0242)	(team, 0.0121)	(player, 0.0108)	(soccer, 0.0104)	(China, 0.0093)	(club, 0.0091)	(Action, 0.00053)
$\vec{{W^{\prime }}_{2}}$	(entertainment, 0.0081)	(Sina, 0.0078)	(reporter, 0.0063)	(Beijing, 0.0048)	(film, 0.0046)	(time, 0.0041)	(Hype, 0.00038)
$\vec{{W^{\prime }}_{3}}$	(furniture, 0.011)	(product, 0.0097)	(furniture, 0.0094)	(design, 0.0093)	(enterprise, 0.0088)	(space, 0.0079)	(Serve, 0.00043)
$\vec{{W^{\prime }}_{4}}$	(home-field, 0.0173)	(winner lottery, 0.0161)	(number, 0.0158)	(current period, 0.0153)	(draw up, 0.0147)	(recommend, 0.0140)	(Trend, 0.00059)
$\vec{{W^{\prime }}_{13}}$	(company, 0.0173)	(market, 0.0162)	(comment, 0.0129)	(billion, 0.0093)	(China, 0.0078)	(increase, 0.0077)	(Employment, 0.00055)
$\vec{{W^{\prime }}_{14}}$	(fund, 0.0701)	(market, 0.0218)	(company, 0.0168)	(invest, 0.0140)	(future, 0.0111)	(rise, 0.0082)	(Settlement, 0.00062)

**Fig 4:**
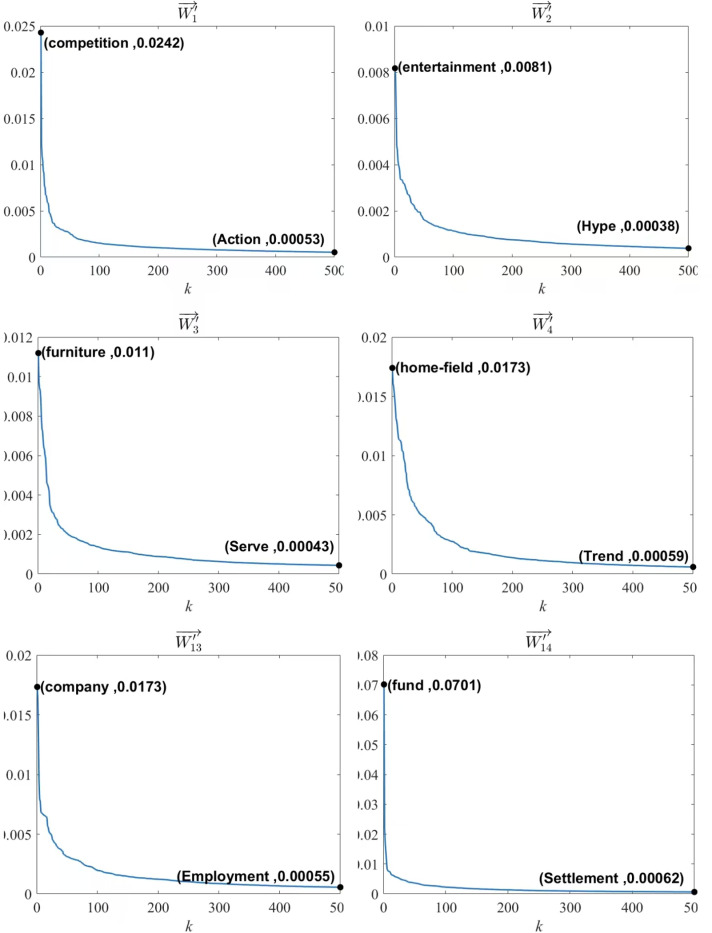
Distribution of Category Feature Representation Weights in Partial Category Feature Vectors.

In Fig 4, six out of the 14 $\vec{{W^{\prime }}_{i}}$ vectors are selected (Sports, Entertainment, Furniture, Lottery, Stock, Finance) to visualize their distribution. Here, the horizontal axis *k* represents the index of the term element in the category feature vector, and the vertical axis i represents the ATF-DF value of the *k* -th term in that category feature vector. Additionally, Fig 4 identifies the terms with the maximum and minimum category feature representation weights among the top 500 terms for the first six $\vec{{W^{\prime }}_{i}}$ vectors, along with their respective weights.

***Results Analysis***: Analysis of the data in Table 3 and Fig 4 is as follows:

The terms included in the category feature vectors are strongly related to the text categories. For instance, in the Sports category, terms such as “match,” “team,” and “player” all exhibit a strong relevance to sports.

The distribution of $w_{ik}$ values across different category feature vectors varies, and the characteristics of $w_{ik}$ distribution align with the linguistic features of different text categories.

For example, for $\vec{{W^{\prime }}_{2}}$ (Entertainment category), 97% of $w_{ik}$ values are concentrated between 0 and 0.005;in contrast, $w_{ik}$ values for $\vec{{W^{\prime }}_{14}}$ (Finance category) are unevenly distributed between 0 and 0.07.

**Fig 5:**
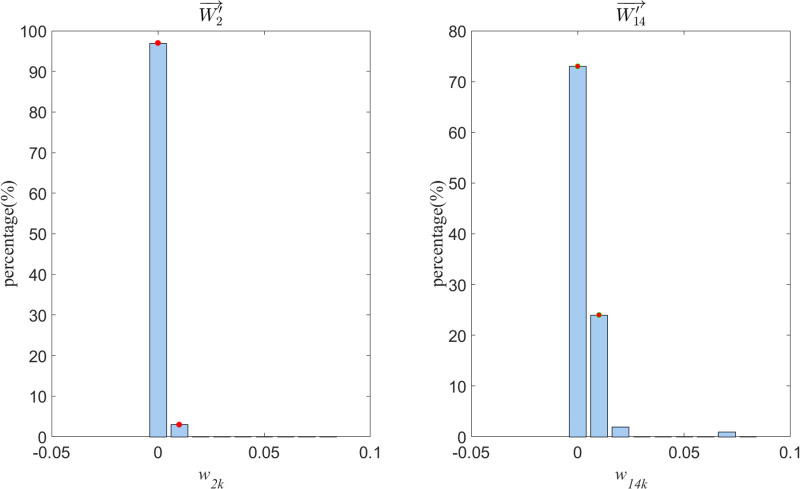
Weight Distribution of Feature Words for Two Categories.

To further analyze, Fig 5 illustrates the distribution of category feature representation weight values for terms in $\vec{{W^{\prime }}_{2}}$ and $\vec{{W^{\prime }}_{14}}$. Here, the horizontal axis $w_{2k}$ and $w_{4k}$ represent the range intervals of the category feature representation weight values for all terms in $\vec{{W^{\prime }}_{2}}$ and $\vec{{W^{\prime }}_{14}}$, respectively, and the vertical axis (percentage %) indicates the proportion of $w_{ik}$ values in that interval range, expressed as a percentage.

As can be seen from Fig 5, for $\vec{{W^{\prime }}_{2}}$, due to the broad range of topics covered by entertainment news corpora, the texts encompass a wide variety of terms. For $w_{ik}$, there is a characteristic of relatively small numerical differences, with 97% of $w_{ik}$ being concentrated between 0 and 0.005, indicating that the vast majority of terms, when viewed individually, do not have a prominent categorical feature representation weight. As for $\vec{{W^{\prime }}_{14}}$, the terms are more focused on common Finance professional jargon, hence when calculating $w_{ik}$, the $w_{ik}$ of Finance jargon is higher, and the $w_{ik}$ of other terms is lower, resulting in an uneven distribution of $w_{ik}$ between 0 and 0.07.

Overall, due to the inherent differences in text categories, there is a variation in the distribution of terms within the corpus, which is precisely the expected effect that the ATF-DF algorithm is designed to achieve.

In summary, based on the analysis of distribution characteristics, it can be concluded that the results and distribution characteristics of the category feature vectors extracted by ATF-DF are in line with the expected design of the ATF-DF algorithm.

#### 3.4.4 Sub-experiment 2: comparative experiment of ATF-DF and TF-IDF on baseline algorithms and result analysis.

***Parameter Settings for Baseline Algorithms:*** The TF-IDF-KNN algorithm is implemented using the Feature extraction and the API interface of sklearn.neighbors.KNeighborsClassifier from the machine learning toolkit scikit-learn. The ATF-DF-KNN algorithm modifies the aforementioned code by replacing the text feature extraction calculation with ATF-DF.

The TF-IDF-NBM algorithm also utilizes the scikit-learn library, implemented by invoking the sklearn.naive_bayes.MultinomialNB. The ATF-DF-NBM algorithm modifies the aforementioned code by replacing the text feature extraction calculation with the ATF-DF method.

The key parameter settings of the two baseline algorithms are shown in Table 4.

**Table 4:** Main Experimental Parameters of Baseline Algorithms.

TF-IDF-KNN	TF-IDF-NBM
K = 3	alpha = 1.0
weights = ‘uniform’	fit_prior=True
algorithm = ‘auto’	class_prior=None
leaf_size = 30	verbose = 0
p = 2	epsilon = 1e-10

***Evaluation Metrics:*** The metrics precision, recall, and the F1 score are utilized to assess the algorithms, with their respective computational equations provided in equations (9), (10), and (11).

Precision: Refers to the proportion of samples that are truly positive among those classified as positive by the classifier. The computational equation is given by:


$$\text{Precision=}\frac{\text{TP}}{\text{TP+FP}}$$ (9)

In this context, True Positive (TP): The number of samples that actually belong to the positive class and are correctly predicted as positive by the model.False Negative (FN): The number of samples that actually belong to the positive class but are incorrectly predicted as negative by the model.

Recall: Indicates the proportion of all actual positive samples that have been correctly identified. The computational equation is given by:


$$\text{Recall=}\frac{\text{TP}}{\text{TP+FN}}$$ (10)

Where the meanings of TP and FN are as defined in equation (11).

F1 Score: A comprehensive measure that takes into account both precision and recall, it is the harmonic mean of precision and recall. The computational equation is given by:


$$\text{F1}=2\cdot \frac{\text{Precision}\cdot \text{Recall}}{\text{Precision+Recall}}$$ (11)

***Experimental Results***: The comparative experimental calculations of ATF-DF-KNN and TF-IDF-KNN are presented in Table 5, while the comparative experimental calculations of ATF-DF-NBM and TF-IDF-NBM are shown in Table 6.

**Table 5:** Comparison Table of ATF-DF-KNN and TF-IDF-KNN.

*i*	Precision		$\alpha_{1}$	Recall		$\alpha_{2}$	F1		$\alpha_{3}$
	A1	T1		A1	T1		A1	T1	
1	0.89	0.82	8.06%	0.63	0.84	-25.00%	0.76	0.83	-8.67%
2	0.78	0.64	21.44%	0.89	0.70	27.57%	0.84	0.67	24.64%
3	0.43	0.22	95.86%	0.79	0.29	171.03%	0.61	0.26	138.61%
4	0.84	0.89	-5.39%	0.92	0.75	22.93%	0.88	0.82	7.56%
5	0.83	0.85	-2.69%	0.58	0.67	-13.58%	0.70	0.76	-7.49%
6	0.66	0.62	6.90%	0.69	0.34	101.76%	0.67	0.48	40.50%
7	0.78	0.23	241.22%	0.77	0.74	4.46%	0.78	0.49	60.60%
8	0.74	0.46	59.91%	0.57	0.39	46.92%	0.65	0.43	53.95%
9	0.88	0.86	1.87%	0.96	0.76	26.58%	0.92	0.81	13.46%
10	0.87	0.71	22.77%	0.90	0.56	61.43%	0.89	0.64	39.82%
11	0.69	0.62	10.65%	0.67	0.56	20.18%	0.68	0.59	15.17%
12	0.73	0.86	-15.63%	0.74	0.64	16.09%	0.73	0.75	-2.09%
13	0.82	0.80	2.96%	0.69	0.40	71.75%	0.76	0.60	25.89%
14	0.66	0.73	-10.08%	0.49	0.35	40.29%	0.57	0.54	6.24%
**Average value**	**0.76**	**0.67**	**13.71%**	**0.74**	**0.57**	**28.94%**	**0.75**	**0.62**	**20.74%**

**Table 6:** Comparison Table of ATF-DF-NBM and TF-IDF-NBM.

C	Precision		$\alpha_{1}$	Recall		$\alpha_{2}$	F1		$\alpha_{3}$
	A2	T2		A2	T2		A2	T2	
1	0.68	0.89	-23.12%	0.87	0.91	-4.29%	0.78	0.90	-13.60%
2	0.83	0.85	-2.51%	0.85	0.76	11.97%	0.84	0.81	4.32%
3	0.90	0.56	60.08%	0.71	0.26	173.08%	0.80	0.41	95.91%
4	0.91	0.9	1.08%	0.91	0.79	14.81%	0.91	0.85	7.50%
5	0.63	0.28	123.25%	0.86	0.92	-6.30%	0.74	0.60	23.92%
6	0.67	0.82	-18.10%	0.81	0.72	13.06%	0.74	0.77	-3.53%
7	0.84	0.66	26.68%	0.66	0.35	89.43%	0.75	0.51	48.42%
8	0.68	0.65	4.89%	0.74	0.44	67.50%	0.71	0.55	30.16%
9	0.82	0.93	-11.74%	0.76	0.79	-4.30%	0.79	0.86	-8.32%
10	0.83	0.85	-1.99%	0.61	0.62	-1.77%	0.72	0.74	-1.90%
11	0.69	0.67	3.22%	0.72	0.7	3.14%	0.71	0.69	3.18%
12	0.66	0.84	-21.66%	0.72	0.79	-9.37%	0.69	0.82	-15.70%
13	0.72	0.85	-14.79%	0.70	0.81	-13.09%	0.71	0.83	-13.96%
14	0.80	0.72	11.11%	0.55	0.69	-20.00%	0.68	0.71	-4.11%
**Average value**	**0.76**	**0.75**	**1.83%**	**0.75**	**0.68**	**9.68%**	**0.75**	**0.72**	**5.57%**

In Table 5, the algorithms ATF-DF-KNN and TF-IDF-KNN are denoted by A1 and T1, respectively, and in Table 6, the algorithms ATF-DF-NBM and TF-IDF-NBM are denoted by A2 and T2, respectively. The specific computational equations are as follows:


$$\alpha_{m}=\pm \frac{\left|S_{m}-Y_{m}\right|}{Y_{m}}\cdot 100\%$$ (12)

In equation (12), $\alpha_{m}$ it denotes the percentage increase or decrease of the $\alpha_{m}$ ATF-DF-KNN algorithm compared to the TF-IDF-KNN algorithm, where $S_{m}$ and $Y_{m}$$\left(m\in \left[1,3\right]\right)$ represent the respective metric values for the ATF-DF-KNN and TF-IDF-KNN algorithms. Here, *m* equals 1 for the Precision metric, *m* equals 2 for the Recall metric, and *m* equals 3 for the F1 metric.

In Table 6, the relevant values in equation (12) are substituted with the experimental outcomes of the ATF-DF-NBM and TF-IDF-NBM algorithms, with the interpretation of the symbols being analogous.

***Analysis of Experimental Results:*** From the experimental results shown in Table 5, it can be observed that the F1-score of the ATF-DF-KNN algorithm in the “Furniture” category has increased by 138.61%, which is a significant improvement. This enhancement is primarily attributed to the ATF-DF algorithm’s accurate capture of category-specific features during the feature vector extraction stage.Specifically, in the “Furniture” category, keywords such as “furniture” and “design” are relatively concentrated, enabling the text classifier to more accurately distinguish this category. In contrast, the improvement in the F1-score for the “Finance” category is only 6.24%, possibly due to the broader distribution of keywords in this category, such as “fund” and “market” which have similar frequencies, thus increasing the classification difficulty. This indicates that the ATF-DF algorithm still has room for improvement when dealing with categories that exhibit low intra-class text similarity.

According to the experimental results in Table 6, the ATF-DF-NBM algorithm achieves an average improvement of 1.83% in the Precision metric and outperforms the TF-IDF-NBM algorithm in nearly half of the text categories. For example, in the “Real Estate” category, the precision of ATF-DF-NBM improves by 123.25%, demonstrating that the ATF-DF algorithm effectively captures distinctive feature words within this category, such as “property” and “apartment type,” thereby enhancing classification accuracy.In contrast, the improvement in the “Lottery” category’s Precision is relatively modest, at only 0.64%, likely due to the greater diversity of terms in this category, such as “home team” and “team,” which makes it challenging to distinctly differentiate feature vectors. The trends observed in Recall and F1-score performance align with these observations.

### 3.5 Experimental conclusions of the ATF-DF algorithm

The following conclusions can be drawn from the research presented in Chapter 2:

(1) The ATF-DF algorithm can accurately extract text category features using a “forward-thinking” frequency statistical approach.Experimental results indicate that ATF-DF outperforms traditional algorithms in terms of classification accuracy, recall, and F1-score, validating its practical application value.
(2) Compared to TF-IDF, ATF-DF demonstrates stronger category-awareness, enabling the precise extraction of feature terms through category-specific information.While TF-IDF focuses on global features and overlooks the distribution of terms within categories, ATF-DF significantly enhances classification accuracy by leveraging category-level term frequency calculations.
(3) ATF-DF more accurately reflects text category characteristics, improving text classification performance.It demonstrates substantial potential and value for practical applications in large-scale text classification tasks.

## 4 ATF-DF-WA algorithm

### 4.1 Algorithm concept

From the perspective of energy vibration, each character and word in the text can be regarded as a pulsating energy symbol. A piece of text can be seen as a two-dimensional, three-dimensional, or even multidimensional waveform diagram, a piece of music, or an interesting image or video, etc. similar to a piece of music or a visual image.In the research of this paper, since the ATF-DF algorithm only retains the frequency characteristics, the calculated class characteristic vector can be transformed into a two-dimensional waveform. Therefore, it is entirely feasible to design it is practical to design a new algorithm that fully utilizes the mature theories and tools of wavelet analysis for text classification calculations. Based on the aforementioned ideas, this paper proposes a new text classification algorithm.

Specifically, the approach and main steps of this new algorithm are:

(1) The class feature vectors obtained based on the ATF-DF algorithm are transformed into typical feature vectors for text categories (referred to as class-typical feature vectors), which are then used to generate the feature vector for the text to be classified.
(2) These feature vectors are converted into waveforms, and through wavelet analysis, the class-typical feature layer waveform, representing the characteristics of a specific text category, and the feature layer waveform of the text to be classified, representing its category characteristics, are obtained.
(3) Calculate the waveform similarity for the aforementioned feature layer waveforms, and complete text classification based on the calculation results.

**Fig 6:**
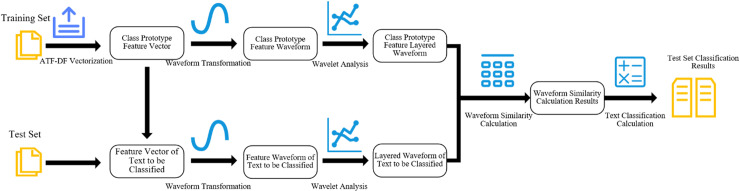
Illustrative diagram of the ATF-DF-WA algorithm.

The aforementioned new algorithm, being based on ATF-DF and further utilizing Wavelet Analysis (WA), is thus named the ATF-DF-WA algorithm. The conceptual diagram of its algorithmic approach is illustrated in Fig 6.

### 4.2 The theory and procedure of the ATF-DF-WA algorithm

**Fig 7:**
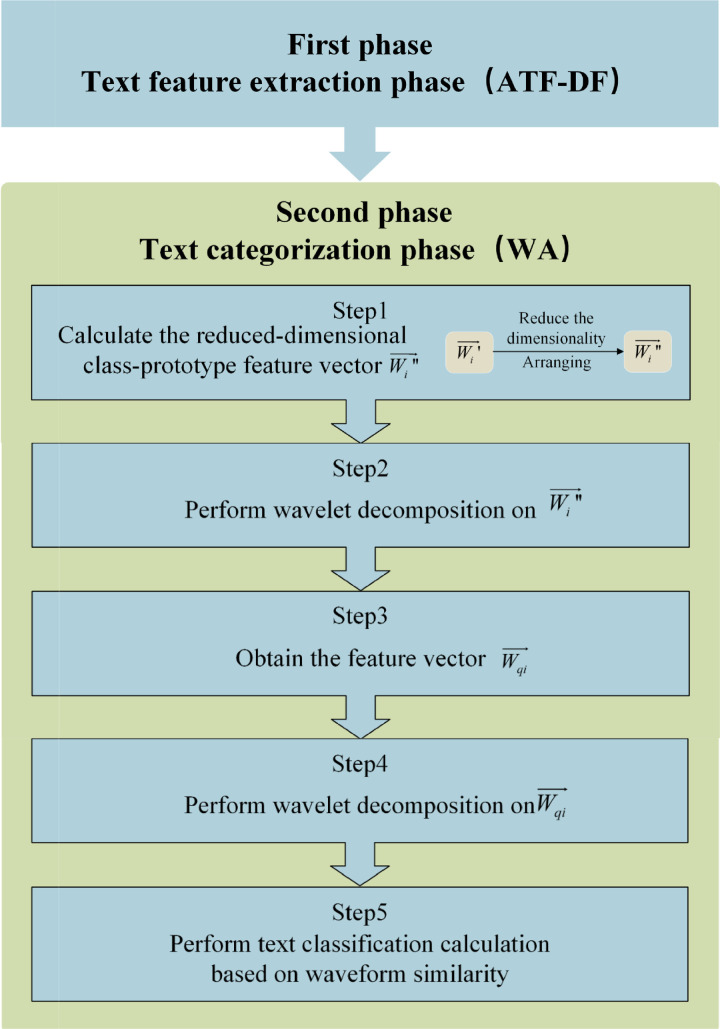
Computational Phases and Steps of the ATF-DF-WA Algorithm.

**Fig 8:**
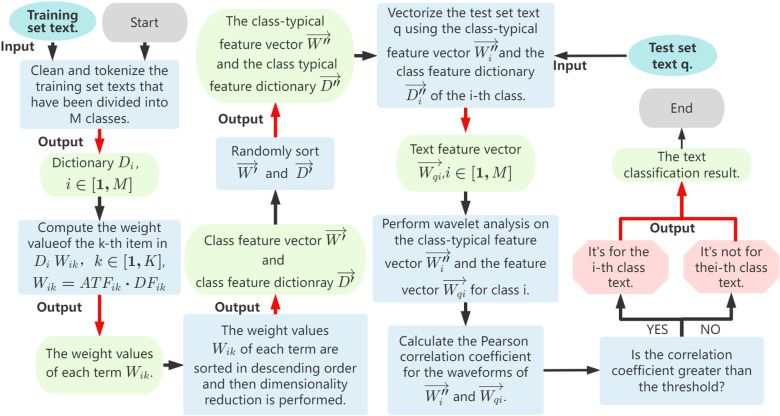
Flowchart of the ATF-DF-WA Algorithm.

Specifically, the ATF-DF-WA algorithm can be divided into two phases: the feature extraction phase and the text classification phase, as illustrated in Fig 7. Fig 8 further elaborates on the detailed process of the algorithm.

Specifically, after the completion of the first phase (the specific steps of which are described in the aforementioned ATF-DF algorithm), the steps of the second phase, as depicted in Fig 8, are as follows:

STEP 1: Perform dimensionality reduction on $\vec{{W^{\prime }}_{i}}$ followed by a random permutation to obtain the class-typical feature vectors Wi''⇀.

Upon completion of the first phase, two issues require resolution:

(1) $\vec{{W^{\prime }}_{i}}$ represents a high-dimensional vector. In order to reduce computational complexity, there arises the issue of dimensionality reduction, involving the identification of its optimal dimensions that effectively capture the essential information while minimizing the complexity.
(2) Different wavelet basis functions, when applied to $\vec{{W^{\prime }}_{i}}$, exhibit variations in the effectiveness of feature extraction, thus presenting the issue of determining the most optimal wavelet basis.

In this paper, the resolution of the two aforementioned issues is accomplished through a unified computational process. Assuming the optimal dimensionality for a particular text category is $K^{'}$, the process of determining $K^{'}$ and the optimal wavelet basis involves the following steps:

On the training dataset, a certain step size is selected to perform dimensionality reduction on $\vec{{W^{\prime }}_{i}}$. Starting from the last term, W is reduced in dimensions by discarding a number of terms equal to the step size, effectively truncating $\vec{{W^{\prime }}_{i}}$ into vectors of different dimensions. Subsequently, for each dimension, a random function is applied to perform shuffling of the terms.

Twelve commonly used wavelet basis functions (‘db2’, ‘db4’, ‘dmey’, ‘haar’, ‘sym2’, ‘sym8’, ‘coif2’, ‘coif4’, ‘bior3.1’, ‘bior5.5’, ‘rbio3.1’, ‘rbio5.5’) are employed to perform text classification calculations. The dimension $K^{'}$ with the highest Precision is chosen as the optimal dimension. In the case of identical Precision results, the lower dimensional value of $K^{'}$ is selected.

The wavelet basis used when Precision is at its highest is also selected as the optimal wavelet basis.

The equation for calculating Precision involved in the aforementioned process is shown in equation (9).

To provide a clear exposition of the details, six additional points are added to elucidate the aforementioned calculation process:

The reason for sorting terms based on ATF-DF weights and then truncating from the last term backward is to retain terms in $\vec{{W^{\prime }}_{i}}$ with higher ATF-DF values, as the preserved terms have better text category differentiation capabilities.

The vectors are truncated to different dimensions and then randomly shuffled again because if they were kept in descending order, the waveforms of $\vec{{W^{\prime }}_{i}}$ would all be monotonically decreasing. This would cause the class typical feature layer waveforms and the feature layer waveforms of the text to be similar in distribution after subsequent wavelet decomposition, leading to poor performance in waveform similarity calculation.

Throughout the computational process mentioned above, the text classification algorithm adopted is the ATF-DF-WA algorithm proposed in this paper.

The theoretical explanation for selecting different wavelet bases for different text categories is as follows: Due to significant differences in the feature distribution across different text categories, it is crucial to choose a wavelet base that matches the category characteristics. Wavelet analysis decomposes text features, and different wavelet bases can better adapt to the characteristics of various texts. For example, sports texts may contain more proper nouns and key terms, while entertainment texts may include more emotional and dynamic expressions. Selecting the appropriate wavelet base can more effectively capture these feature differences, thereby improving classification performance.

The theoretical explanation for the dimensionality reduction strategy is as follows: In text feature vector processing, high-dimensional feature spaces increase the computational complexity of classification algorithms and may introduce redundant features and noise, leading to decreased classification performance. Dimensionality reduction removes low-weight and redundant features, preserving the primary category characteristic information. Wavelet analysis theory indicates that the main energy of a signal is typically concentrated in a few features. Therefore, dimensionality reduction not only enhances computational efficiency but also improves the robustness and performance of the classification model.

For a better understanding and a more intuitive explanation, examples of the calculation of the optimal dimension and the best wavelet basis for each text category are provided in the experimental section of this paper.

After obtaining $K^{'}$ and determining the optimal dimension $K^{'}$ for it, the corresponding $\vec{{W^{\prime }}_{i}}$ with this optimal dimensionality becomes the class-typical feature vectors, denoted as Wi''⇀. Mathematically, this can be expressed as:


$$\vec{W_{i}^{''}}=\left[\begin{array}{c} W_{1}\\ W_{2}\\ \vdots \\ W_{i}\\ \vdots \\ W_{K^{'}} \end{array}\right]$$ (13)

Where $W_{i}(i\in \left[1,K^{'}\right])$ is a row vector, which can be represented as:


$$W_{i}=\left[t_{ik}w_{ik}\right]$$ (14)

The complete expression of the row vector elements for Wi''⇀ is as follows:


$$\vec{W_{i}^{''}}=\left[\begin{array}{c} t_{i1}w_{i1}\\ t_{i1}w_{i2}\\ \begin{array}{c} \vdots \\ t_{ik}w_{ik}\\ \vdots \end{array}\\ t_{iK^{'}}w_{iK^{'}} \end{array}\right]$$ (15)

STEP 2: Obtain the Class-Typical Feature Layer Waveform by Wavelet Decomposition of Wi''⇀

Decompose Wi''⇀ using the optimal wavelet basis, and select the first-level wavelet as the class-typical feature wavelet of Wi''⇀. There are two reasons for choosing the first-level wavelet:

(1) Observation: It was observed during the experiments that the first-level wavelet retains richer details of waveform features compared to other levels.
(2) Calculation: Choosing the first-level and other-level waveforms as class-typical feature waveforms, calculations are performed on the training dataset. The results indicate that the average precision of classification is optimal for the first level. Detailed experimental data can be found in the experimental section of this paper.)

STEP3: The feature vector $\vec{W_{qi}}$ for *q* is computed.)

Tokenize and preprocess *q*, then calculate the feature vector $\vec{W_{qi}}$ based on Wi''⇀. The specific calculation process is as follows:

Iterate through *k* from 1 to $K^{'}$, comparing each term of Wi''⇀ (denoted as $t_{ik}$) with all terms in *q*. If a term in *q* matches $t_{ik}$ from Wi''⇀ (where $t_{ik}$ represents the *k* -th term in Wi''⇀), then compute the *k* -th element value of $\vec{W_{qi}}$ according to (16); otherwise, set the *k* -th element value to 0.


$$w_{qik}=TF_{qik}\cdot DF_{ik}$$ (16)

Where $w_{qik}$ represents the *k* -th element value of $\vec{W_{qi}}$, $TF_{qik}$ represents the term frequency of $t_{ik}$ in the corpus *q*, and $DF_{ik}$ is the DF value of $t_{ik}$ in Wi''⇀.

**Fig 9:**
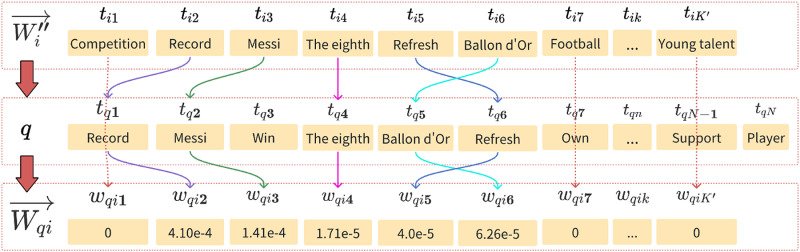
Example of the specific process for generating $\vec{W_{qi}}$.

As an illustrative example, Fig 9 demonstrates the specific process of generating the feature vector $\vec{W_{qi}}$ corresponding to *q* using a particular Wi''⇀. In Fig 9, assuming there are *N* terms in $\vec{W_{qi}}$, let’s denote the *k* -th element of $\vec{W_{qi}}$ as $w_{qik}$, and suppose *N* is greater than *K*.)

The specific calculation process for this example is as follows: starting from the first element of Wi''⇀, since the term $t_{i1}$ “competition” cannot be found in any terms of *q*, the value of $w_{qi1}$ is set to 0. The second element of Wi''⇀, $t_{i2}$ “record”, exactly matches the first element $t_{q1}$ in *q* (indicating $t_{i2}$ can be found in the elements of *q*). According to (16), the second element of $\vec{W_{qi}}$, $w_{qi2}$, is calculated as $4.10\times 10^{-4}$. The same procedure is applied for similar cases for other elements. Following the above rules, the process continues from the first element of Wi''⇀ until the $K^{'}$ -th element is processed, resulting in $\vec{W_{qi}}$ with the same dimension $K^{'}$.

STEP 4: Perform a wavelet decomposition on $\vec{W_{qi}}$ to obtain the characteristic waveform of the feature layer *q*.

Decompose $\vec{W_{qi}}$ using the optimal wavelet basis for the text category, obtaining the first-level wavelet form for the sample to be classified, which is the feature-level waveform for Wi''⇀.

STEP 5: Utilize waveform similarity to accomplish text classification.

Utilize the Pearson correlation coefficient shown in (5) to calculate the similarity between the feature-level waveform of *q* and the class-typical waveform of Wi''⇀, and complete the text classification. The specific process is as follows:

Iterate A from 1 to M, conducting the following computations:

(1) Based on the characteristic waveform of the feature layer Wi''⇀ corresponding to *q*, use equation (16) to calculate the similarity $r_{i}$ between this feature layer waveform and the *i* -th class-representative characteristic waveform.
(2) If the calculated result exceeds a predefined threshold, *q* is classified as belonging to the *i* -th category, and the calculation ends.
(3) Otherwise, continue generating the feature-level waveform of *q* corresponding to the next feature layer waveform(Corresponding the next class-typical feature vector), and calculate the similarity between this feature-level waveform and the next class-typical feature-level waveform. Repeat this process until the similarity exceeds the threshold for successful classification.
(4) If none of the calculated results exceed the predefined threshold, take the index *i* corresponding to the maximum value of $r_{i}$ and classify *q* as belonging to the *i* -th class of text.

The reason for selecting the Pearson correlation coefficient to calculate the similarity of waveforms in this paper is: ① The Pearson correlation coefficient is suitable for discrete waveform data (waveform information sets that are discontinuous in time or space and exist in the form of discrete points). ② Although there are other methods that can also be applied to discrete data, the Pearson correlation coefficient is more intuitive and easier to interpret when assessing similarity. Considering that the ATF-DF-WA algorithm is a shallow learning algorithm, in order to retain its interpretable advantage, taking into account the characteristics of the data and the research objectives, the Pearson correlation coefficient method has become the choice of this paper.

### 4.3 ATF-DF-WA algorithm experiments

#### 4.3.1 Description of experimental dataset and environmental parameters.

To fully validate the text classification performance of ATF-DF-WA, this experimental phase selected three Chinese datasets on the experimental dataset, namely THUCHNews described in section 3.4.2, Sogou Chinese Corpus (Sogou) [32](download link: http://b.mtw.so/5W8eMF), and Chinese News Text Categorization (CNTC) [33] (download link: https://aistudio.baidu.com/datasetdetail/125160/0).

The Sogou corpus is a widely used Chinese text classification dataset, sourced from Sogou News, and contains 17,910 text samples. The specific categories and the number of texts in each category are shown in Table 7.

**Table 7:** Data Categories of the Sogou Corpus.

name	Category	Number of text	name	Category	Number of text
C000008	Finance	1990	C000020	Education	1990
C000010	IT	1990	C000022	Hire	1990
C000013	Health	1990	C000023	Culture	1990
C000014	Sports	1990	C000024	Military Affairs	1990
C000016	Torism	1990			

The CNTC corpus is a public dataset on the Baidu PaddlePaddle AI Studio platform, containing 668,854 text samples. The specific categories and the number of texts in each category are shown in Table 8.

**Table 8:** Data Categories of the CNTC News Corpus.

Category	Number of text	Category	Number of text
Real-Estate	16040	Entertainment	74105
Current Affairs	50468	Furniture	26068
Sports	105283	Education	33548
Finance	29678	Social	40679
Technology	130343	Constellation	2862
Stock	123518	Games	19498
Lottery	6070	Fashion	10694

The reasons for selecting these two corpora are as follows: (1) Both corpora cover multiple news categories, providing comprehensive support for text classification tasks across different fields, making them suitable for multi-scenario text mining research. (2) The Sogou corpus has a balanced distribution of text samples across categories, while the CNTC corpus exhibits significant class imbalance. By combining these two datasets, which complement each other in terms of sample balance and imbalance, the study can offer comprehensive data support for addressing the issues of balanced and imbalanced category samples, forming an ideal foundation for comparative experimental research.

For all the corpora, 80% of the data is selected as the training dataset, and 20% is used as the test dataset.

Other computational environment parameters are the same as those in Section 3.4.2.

#### 4.3.2 Sub-experiment 1: step-by-step calculation results and analysis of the ATF-DF-WA algorithm.

***Optimal Dimension*** $K^{'}$ ***and Best Wavelet Basis Calculation Results:*** In this experiment, the THUCHNews dataset is used, for the 14 $\vec{W_{i}^{'}}$ in the training dataset, a step size of 1000 is used to calculate their optimal dimension $K^{'}$ and the best wavelet basis. The calculation steps are described in STEP1 of Section 4.2, with results presented in Table 9.

**Table 9:** Optimal dimension and optimal wavelet base of 14 categories.

*i*	$K^{'}$	Optimal wavelet basis	Precision
1	7000	sym8	100.00%
2	9000	sym8	100.00%
3	10000	sym8	99.70%
4	15000	sym8	99.90%
5	10000	rbio3.1	100.00%
6	15000	dmey	100.00%
7	10000	sym2	100.00%
8	12000	db2	100.00%
9	12000	db2	99.80%
10	8000	sym2	100.00%
11	11000	rbio3.1	100.00%
12	13000	coif2	100.00%
13	14000	sym8	100.00%
14	14000	sym2	99.20%

In this experiment, the results from Table 7 are also applied to the other two datasets.

Based on the calculation results from Table 7, set all dimensions of $\vec{{W^{\prime }}_{i}}$ to correspond to $K^{'}$, thereby obtaining 14 class-typical feature vectors Wi''⇀, and represent them all using two-dimensional waveform diagrams.

**Fig 10:**
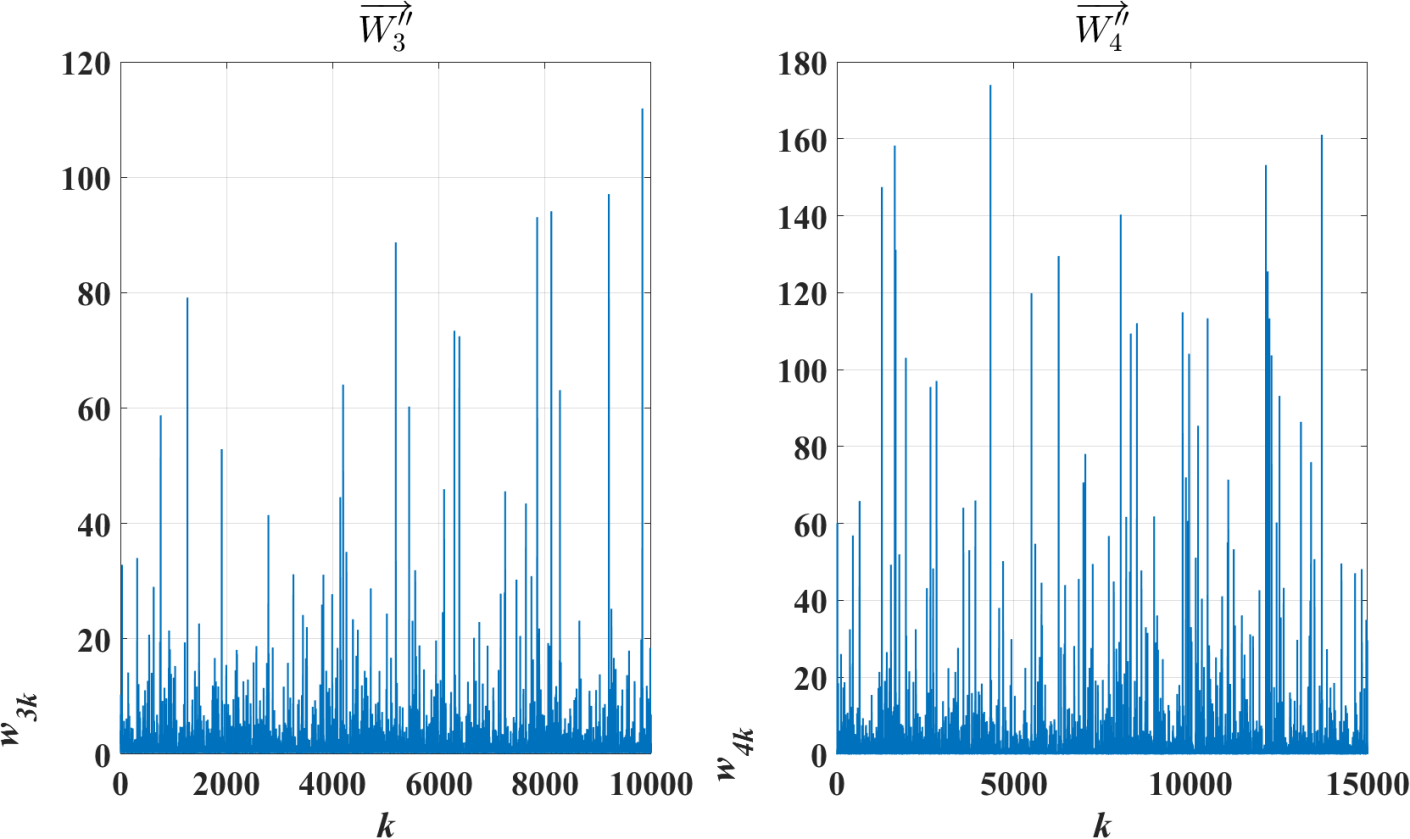
Example of Class-typical Feature Vectors.

As an example, Fig 10 illustrates the waveforms of two class-typical feature vectors, denoted as $\vec{{{W^{\prime }}^{\prime }}_{3}}$ and $\vec{{{W^{\prime }}^{\prime }}_{4}}$. Here, the abscissa corresponds to the sequence number of the vector elements, and the ordinate represents the ATF-DF values of these elements, scaled up by 10,000 times for clarity.

Fig 10 clearly shows that the waveform distribution characteristics of the class-typical feature vectors differ significantly across different text categories.

***Selection Calculation Results of Class Typical Feature Layers:*** In this paper, Wi''⇀ undergoes 14 layers of wavelet analysis, resulting in 14 hierarchical waveforms. However, a specific layer of the waveform needs to be selected as the class-typical feature layer for subsequent computations. The selection process involves the following steps:

Perform a 14-layer wavelet decomposition on Wi''⇀, which has a dimensionality of $K^{'}$, to obtain the waveforms of each layer.

**Fig 11:**
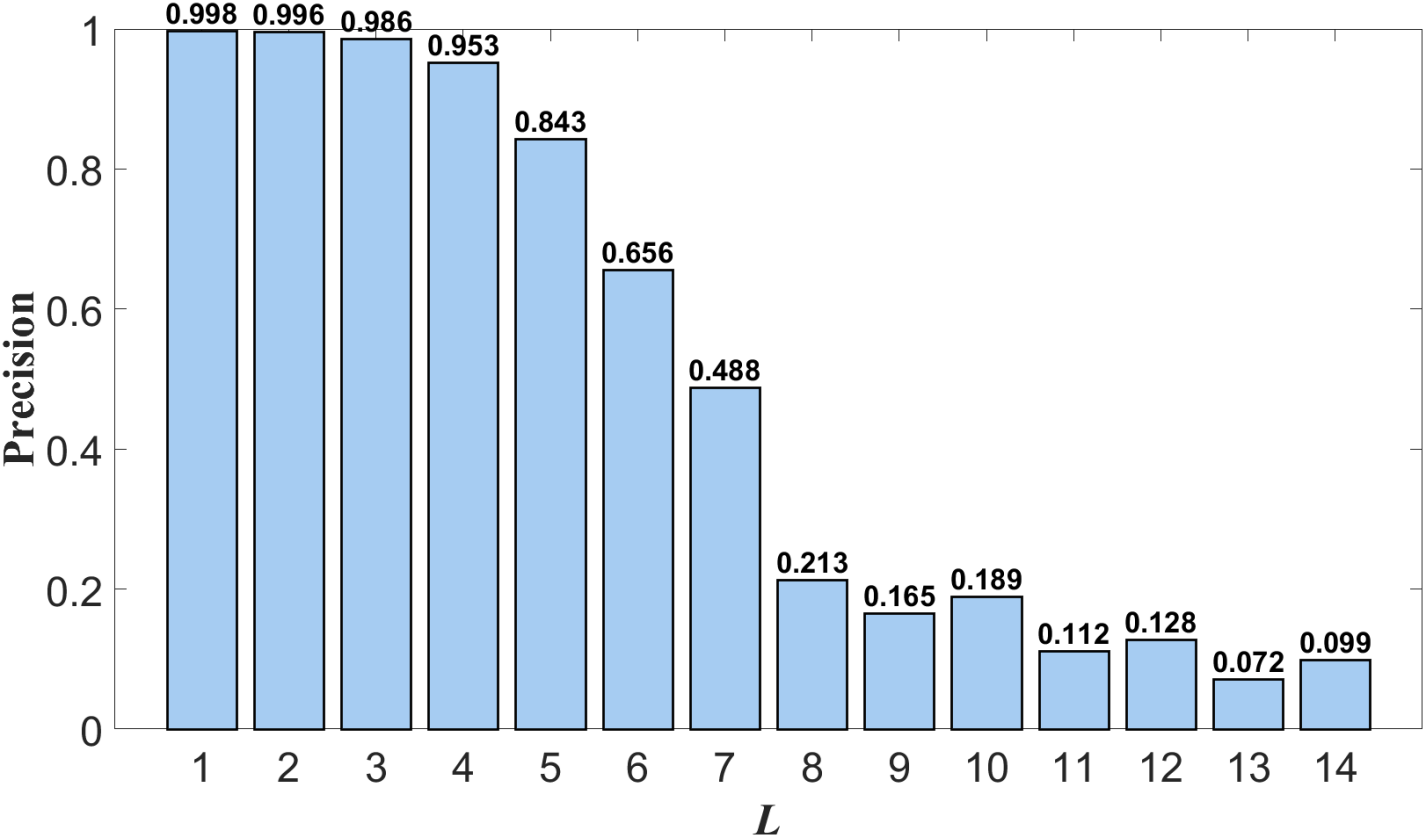
Accuracy of Each Layer.

On the training dataset, each of the 14 layers of waveforms was used sequentially as the class typical feature layer waveform, and text classification was performed using the ATF-DF-WA algorithm proposed in this paper. The results are shown in Fig 11, where the horizontal axis L represents the L-th layer waveform obtained after decomposition, and the vertical axis Precision represents the average classification precision.

The waveform layer with the optimal Precision value is selected as the class-typical feature waveform layer. As can be observed from Fig 11, the first layer waveform exhibits the best experimental Precision value, thus it is chosen as the class-typical feature waveform layer for further computations.

**Fig 12:**
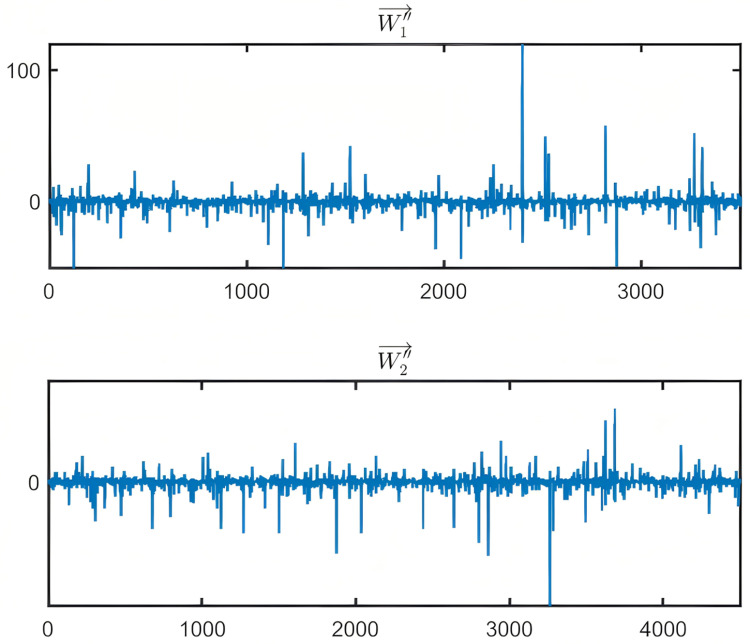
Example of a typical feature layer waveform for Category Wi''⇀.

As an example, Fig 12 shows the waveforms of two typical feature layers of Categories $\vec{{{W^{\prime }}^{\prime }}_{1}}$ and $\vec{{{W^{\prime }}^{\prime }}_{1}}$ obtained after wavelet decomposition. In the Fig, the horizontal axis represents the sequence number of the waveform elements, while the vertical axis represents the amplitude of the waveform elements.

From Fig 12, it can be observed that the typical feature waveform layer of Category Wi''⇀ not only retains rich detail information but also exhibits distinct waveform distribution characteristics.

***The feature vector calculation result of a certain text*** *q* ***to be classified:*** Input the text *q* to be classified from the test dataset, perform word segmentation on the text, and then obtain 14 feature vectors $\vec{W_{qi}}$ (i∈[1,14]) for the test text according to the algorithm described in STEP3 of Section 4.2.

**Fig 13:**
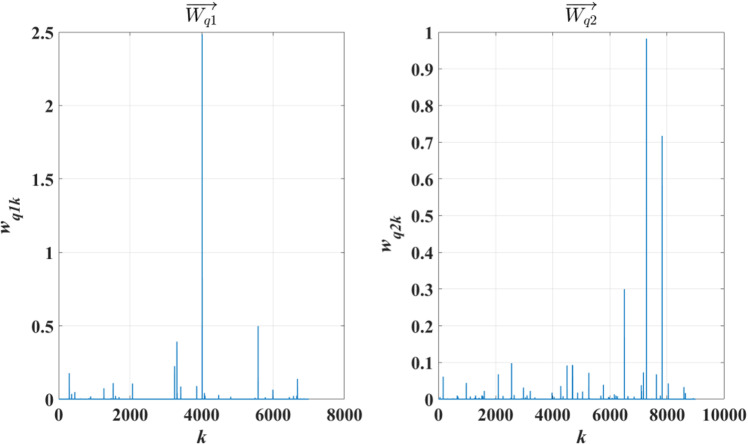
Example of Feature Vectors for Text. *q*.

As an example, Fig 13 shows the $\vec{W_{q1}}$ and $\vec{W_{q2}}$ for a test text *q*, where the horizontal axis represents the index of the vector elements and the vertical axis represents the vector element values amplified by a factor of 10,000.

As seen in Fig 13, the feature vectors of text *q* also exhibit significant differences in their waveform distribution.

***The waveform calculation result of the feature layer for a certain text*** *q* ***to be classified:*** Using the optimal wavelet basis Wi''⇀ corresponding to all $\vec{W_{qi}}$ (i∈[1,14]) of text *q*, perform a 14-level wavelet decomposition according to the algorithm in STEP4 of Section 4.2, and retain the 14 first-level waveforms as the feature layer waveforms of text *q*.

**Fig 14:**
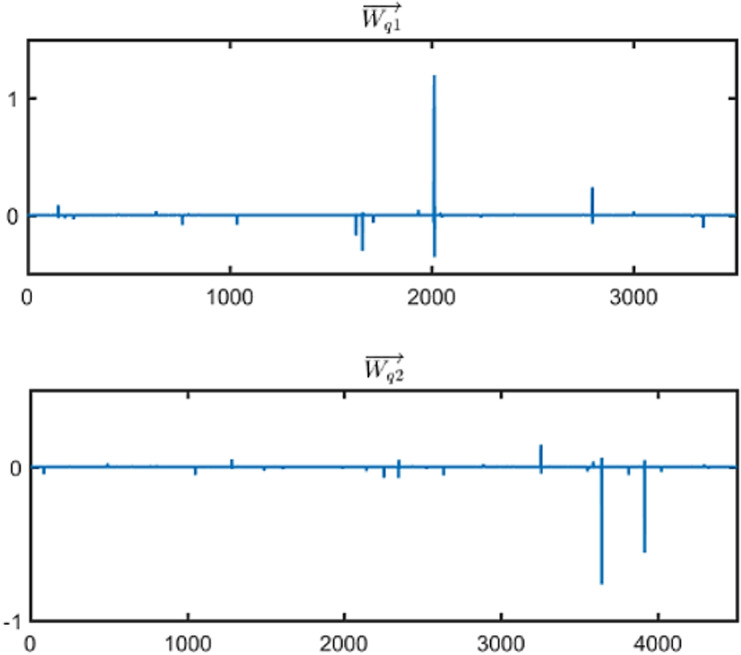
Example of Feature Layer Waveforms for Text. *q*.

As an example, Fig 14 illustrates two feature layer waveforms ($\vec{W_{q1}}$ and $\vec{W_{q2}}$) for text *q*. In the Fig, the horizontal axis represents the index of the waveform elements, and the vertical axis represents the amplitude of the waveform elements.

As shown in Fig 14, the feature layer waveform obtained from the wavelet decomposition of $\vec{W_{qi}}$ exhibits distinct characteristics in its waveform distribution, which aligns with the expectations of the algorithm design.

***The text classification calculation result for a certain text*** *q* ***to be classified:*** The text classification calculation is completed according to the theoretical guidance of STEP5 described in Section 4.2.

As an example, Table 10 presents the calculation results of the similarity $r_{i}$ between the feature layer waveform of a certain text *q* and the class-typical feature layer waveform Wi''⇀.

**Table 10:** Pearson Correlation Coefficient Calculation Results for. *q.*

*i*	$r_{i}$	*i*	$r_{i}$
1	-0.006533291	8	0.015727544
2	-0.011778072	9	0.028964917
3	-0.014670917	10	-0.00090003
4	-0.002466252	11	-0.00685191
5	0.004465182	12	0.00120684
6	-0.012341802	13	-0.004381383
7	0.008387597	14	0.389493928

In the example, it can be seen that $r_{14}$ is greater than the threshold, while the other $r_{i}$ values are below the threshold. Therefore, *q* is classified as the 14th category, which is financial text.

#### 4.3.3 Sub-experiment 2: text classification results and analysis of ATF-DF-WA vs. traditional baseline algorithms.

To extensively validate the text classification performance of ATF-DF-WA, Sub-experiment 2 builds upon Sub-experiment 1 by adding the Sogou and CNTC datasets, in addition to the THUCHNews dataset. Calculations and analyses are then conducted on these three datasets, comparing the results with traditional baseline algorithms.

***Selection of the Baseline Algorithm:*** As mentioned earlier, ATF-DF-WA does not utilize a deep learning framework and belongs to the category of shallow learning algorithms. However, to enable a comprehensive performance evaluation, this paper selects four baseline algorithms: the shallow learning algorithms TF-IDF-KNN and LibSVM, as well as the deep learning algorithms TextCNN [34] and TextRNN [35]. In terms of evaluation metrics, precision, recall, and F1 score are all recorded.

***Baseline Algorithm Parameter Settings:*** The implementation and parameter settings of the TF-IDF-KNN algorithm are consistent with those used in the experiments of Section 3.

The implementation of the LibSVM algorithm uses an open-source library for SVM classification [36].

The implementation of the TextCNN and TextRNN algorithms was built using Keras [37].

Table 11 shows the settings for some key parameters.

**Table 11:** Core Parameter Settings for Baseline Algorithms.

LibSVM	TextCNN	TextRNN
-s svm_type = C-SVC	MAX_SEQUENCE_LENGTH = 1000	Embedding_input_dim = 20000
-t kernel_type = RBF kernel	MAX_NUM_WORDS = 20000	input_length = 1000
-c cost = 1	EMBEDDING_DIM = 128	embedding_dim = 300
-g gamma = 1/n_features	NUM_CLASSES = 14	lstm_units = 128
-m cachesize = 100MB	output_dim = 300	learning_rate = 0.001
degree = 3	kernel_size = 5	Epochs = 20
coef0 = 0.0	activation = ‘relu’	dropout_rate = 0.5

***Experimental Calculation Results:*** Through experiments on the THUCHNews dataset, the evaluation metrics for the classification performance of the five algorithms across 14 categories, including Precision, Recall, and F1 score, are presented in Tables 12, 13, and 14, respectively; Across all three datasets, the average Precision, Recall, and F1 score evaluation metrics for the five algorithms are shown in Tables 15–17.

**Table 12:** Comparison of Precision Values for the Five Algorithms.

Category	① (Our algorithm)	②	$\alpha_{p2}$	③	$\alpha_{p3}$	④	$\alpha_{p4}$	⑤	$\alpha_{p5}$
**Sports**	**1.000**	0.770	29.87%	0.979	2.15%	1.000	0.00%	0.992	0.81%
**Entertainment**	**1.000**	0.630	58.73%	0.936	6.84%	0.983	1.73%	0.957	4.49%
**Furniture**	**0.997**	0.530	88.11%	0.871	14.47%	0.964	3.42%	0.955	4.40%
**Lottery**	**0.999**	0.710	40.70%	/	/	/	/	/	/
**Real-Estate**	**1.000**	0.430	132.56%	0.957	4.49%	0.981	1.94%	0.995	0.50%
**Education**	**1.000**	0.310	222.58%	0.887	12.74%	0.957	4.49%	0.947	5.60%
**Fashion**	**1.000**	0.590	69.49%	0.868	15.21%	0.982	1.83%	0.926	7.99%
**Current Affairs**	**1.000**	0.690	44.93%	0.764	30.89%	0.963	3.84%	0.920	8.70%
**Constellation**	**0.998**	0.820	21.71%	/	/	/	/	/	/
**Games**	**1.000**	0.720	38.89%	0.922	8.46%	0.983	1.73%	0.963	3.84%
**Social**	**1.000**	0.760	31.58%	/	/	/	/	/	/
**Technology**	**1.000**	0.840	19.05%	0.845	18.34%	0.943	6.04%	0.964	3.73%
**Stock**	**1.000**	0.580	72.41%	/	/	/	/	/	/
**Finance**	**0.992**	0.550	80.36%	0.779	27.34%	0.891	11.34%	0.887	11.84%
**Average value**	**0.992**	0.550	80.36%	0.881	12.60%	0.965	2.80%	0.951	4.31%
**Confidence interval**	**[0.996,1.000]**	**[0.498,0.703]**		**[0.835,0.926]**		**[0.945,0.984]**		**[0.930,972]**	

**Table 13:** Comparison of Recall Values for the Five Algorithms.

Category	① (Our algorithm)	②	$\alpha_{z2}$	③	$\alpha_{z3}$	④	$\alpha_{z4}$	⑤	$\alpha_{z5}$
**Sports**	0.992	0.860	15.35%	0.986	0.61%	0.996	-0.40%	0.978	1.43%
**Entertainment**	1	0.910	9.89%	0.966	3.52%	0.990	1.01%	0.968	3.31%
**Furniture**	0.795	0.290	174.14%	0.883	-9.97%	**0.912**	-12.83%	0.822	-3.28%
**Lottery**	**0.997**	0.830	20.12%	/	/	/	/	/	/
**Real-Estate**	**0.997**	0.610	63.44%	0.953	4.62%	0.931	7.09%	0.996	0.10%
**Education**	0.901	0.450	100.22%	0.85	6.00%	**0.959**	-6.05%	0.892	1.01%
**Fashion**	0.994	0.560	77.50%	0.881	12.83%	0.965	3.01%	0.969	2.58%
**Current Affairs**	**0.987**	0.630	56.67%	0.868	13.71%	0.929	6.24%	0.939	5.11%
**Constellation**	**0.999**	0.950	5.16%	/	/	/	/	/	/
**Games**	**0.978**	0.700	39.71%	0.536	82.46%	0.976	0.20%	0.972	0.62%
**Social**	**0.991**	0.460	115.43%	/	/	/	/	/	/
**Technology**	0.965	0.590	63.56%	0.882	9.41%	**0.980**	-1.53%	0.964	0.10%
**Stock**	**0.987**	0.670	47.31%	/	/	/	/	/	/
**Finance**	0.939	0.220	326.82%	0.656	43.14%	**0.998**	-5.91%	0.994	-5.53%
**Average value**	**0.965**	0.624	54.65%	0.846	14.07%	0.964	0.10%	0.949	1.69%
**Confidence interval**	[0.915,0.997]	[0.445,0.726]		[0.755,0.937]		[0.944,0.983]		[0.915,984]	

**Table 14:** Comparison of F1 Score Metrics for the Five Algorithms.

Category	① (Our algorithm)	②	$\alpha_{f2}$	③	$\alpha_{f3}$	④	$\alpha_{f4}$	⑤	$\alpha_{f5}$
**Sports**	0.996	0.810	22.96%	0.983	1.32%	**0.998**	-0.20%	0.985	1.12%
**Entertainment**	**1.000**	0.740	35.14%	0.951	5.15%	0.987	1.32%	0.962	3.95%
**Furniture**	0.885	0.370	139.19%	0.877	0.91%	0.937	-5.55%	0.883	0.23%
**Lottery**	**0.998**	0.770	29.61%	/	/	/	/	/	/
**Real-Estate**	**0.998**	0.500	99.60%	0.955	4.50%	0.955	4.50%	0.996	0.20%
**Education**	0.949	0.370	156.49%	0.868	9.33%	**0.958**	-0.94%	0.919	3.26%
**Fashion**	**0.997**	0.570	74.91%	0.875	13.94%	0.973	2.47%	0.947	5.28%
**Current Affairs**	**0.993**	0.660	50.45%	0.813	22.14%	0.946	4.97%	0.929	6.89%
**Constellation**	**0.999**	0.880	13.52%	/	/	/	/	/	/
**Games**	**0.989**	0.710	39.30%	0.678	45.87%	0.900	9.89%	0.968	2.17%
**Social**	**0.995**	0.570	74.56%	/	/	/	/	/	/
**Technology**	**0.982**	0.690	42.32%	0.863	13.79%	0.961	2.19%	0.964	1.87%
**Stock**	**0.993**	0.620	60.16%	/	/	/	/	/	/
**Finance**	**0.965**	0.310	211.29%	0.713	35.34%	0.942	2.44%	0.937	2.99%
**Average value**	**0.981**	0.610	60.82%	0.858	14.34%	0.956	2.62%	0.949	3.37%
**Confidence interval**	**[0.953,0.999]**	**[0.463,0.689]**		**[0.794,0.921]**		**[0.938,0.973]**		**[0.928,970]**	

**Table 15:** Comparison of Average Precision Values Across Three Different Datasets.

	① (Our algorithm)	②	$\alpha_{p2}$	③	$\alpha_{p3}$	④	$\alpha_{p4}$	⑤	$\alpha_{p5}$
**THUCHNews**	**0.992**	0.550	80.36%	0.881	12.60%	0.965	2.80%	0.951	4.31%
**Sogou**	**1**	0.556	80.00%	0.736	35.95%	0.836	19.68%	0.741	34.93%
**CNTC**	**0.999**	0.658	51.93%	0.802	24.57%	0.963	3.73%	0.908	10.05%
**Average value**	**0.997**	0.588	69.56%	0.806	23.65%	0.921	8.21%	0.867	15.04%

**Table 16:** Comparison of Average Recall Values Across Three Different Datasets.

	① (Our algorithm)	②	$\alpha_{z2}$	③	$\alpha_{z3}$	④	$\alpha_{z4}$	⑤	$\alpha_{z5}$
**THUCHNews**	**0.965**	0.624	54.65%	0.846	14.07%	0.964	0.10%	0.949	1.69%
**Sogou**	**0.983**	0.503	95.34%	0.724	35.72%	0.830	18.46%	0.721	36.35%
**CNTC**	**0.992**	0.553	79.20%	0.688	44.17%	0.964	2.81%	0.911	8.83%
**Average value**	**0.980**	0.560	75.00%	0.753	30.20%	0.919	6.60%	0.860	13.91%

**Table 17:** Comparison of Average F1 Scores Across Three Different Datasets.

	① (Our algorithm)	②	$\alpha_{f2}$	③	$\alpha_{f3}$	④	$\alpha_{f4}$	⑤	$\alpha_{f5}$
**THUCHNews**	**0.981**	0.610	60.82%	0.858	14.34%	0.956	2.62%	0.949	3.37%
**Sogou**	**0.991**	0.512	93.54%	0.728	36.21%	0.830	19.44%	0.720	37.69%
**CNTC**	**0.995**	0.556	79.16%	0.704	41.29%	0.963	3.31%	0.909	9.55%
**Average value**	**0.989**	0.559	76.82%	0.763	29.56%	0.916	7.93%	0.859	15.09%

Some notes on the above table:

(a) In the tables above, ① represents ATF-TF-WA, ② TF-IDF-KNN, ③ LibSVM, ④ TextCNN, and ⑤ TextRNN, The numbers highlighted in bold indicate the highest values among the five algorithms for the respective metrics.
(b) The data in the tables are the results of 20 runs. The results for ①, ②, and ③ are consistent across multiple runs, with negligible random variation, while the results for ④ and ⑤ show slight differences across runs, so the average value is taken.
(c) The calculation formula for $\alpha_{mn}$ in Tables 12–14 is as follows:

$$\alpha_{mn}=\pm \frac{\left|S_{mn}-Y_{mn}\right|}{Y_{mn}}\cdot 100\%$$ (17)

Where $\alpha_{mn}$ represents the percentage increase or decrease of the ATF-DF-WA algorithm compared to the other four algorithms, $S_{mn}$ and $Y_{mn}$ represent the metric values of the ATF-DF-WA algorithm and the other four algorithms, respectively, where: *m* represents the corresponding metric: *p* for the Precision metric, *z* for the Recall metric, and *f* for the F1 score metric.; *n* corresponds to the algorithms as follows: *n* = 2 corresponds to the TF-IDF-KNN algorithm, *n* = 3 corresponds to the LibSVM algorithm, *n* = 4 corresponds to the TextCNN algorithm, and *n* = 5 corresponds to the TextRNN algorithm. In the equation, the “+” and “-” signs on the right indicate that the ATF-DF-WA algorithm’s metric is better or worse than that of the TF-IDF-KNN algorithm, respectively.

(d) The confidence intervals in Tables 12–14 are represented in the form of $\left[CI_{\min},CI_{\max}\right]$, where $CI_{\min}$ denotes the lower bound of the confidence interval, representing the minimum possible value of the population parameter at a given confidence level, and $CI_{\max}$ represents the upper bound, indicating the maximum possible value of the population parameter at the same confidence level.

df=G−1 (18)

$$SE=\frac{s}{\sqrt{G}}$$ (19)

$$s=\sqrt{\frac{\sum {\left(x_{i}-\bar{x}\right)}^{2}}{df}}$$ (20)

$$CI_{\min}=\bar{x}-Z_{df,\beta }\cdot SE$$ (21)

$$CI_{\max}=\bar{x}+Z_{df,\beta }\cdot SE$$ (22)

In Equation (18), df represents the degrees of freedom, and *G* represents the sample size;In Equation (19), SE denotes the standard error, and *s* refers to the sample standard deviation; In Equation (20), $\bar{x}$ represents the sample mean; In Equation (21), $CI_{\min}$ denotes the minimum possible value of the population parameter at a given confidence level, and $Z_{df,\beta }$ represents the significance level *β* (two-tailed) corresponding to df;In Equation (22), $CI_{\max}$ denotes the maximum possible value of the population parameter at the same confidence level, with other parameters having the same meaning as in Equation (21).

According to the t-Distribution Table, when *G* is 14, df =  13, and *β* =  0.05, $Z_{df,\beta }$ the corresponding value is 2.16.

**Fig 15:**
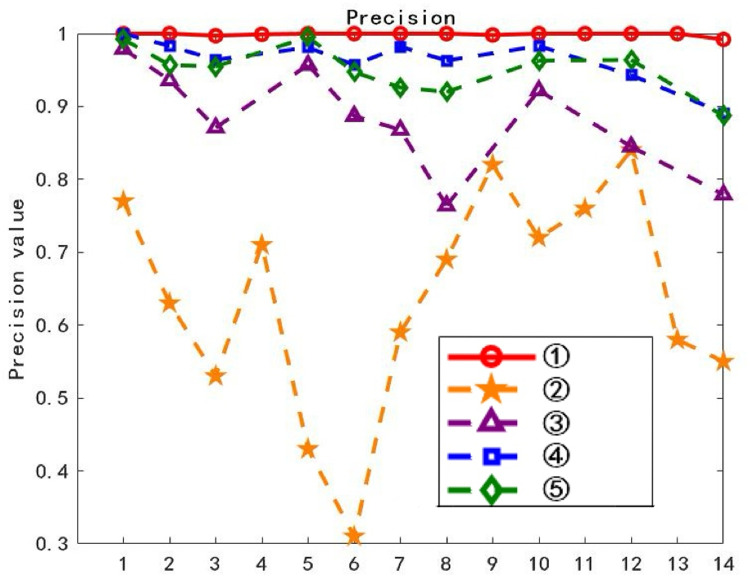
Comparison of Precision Values for the Five Algorithms.

**Fig 16:**
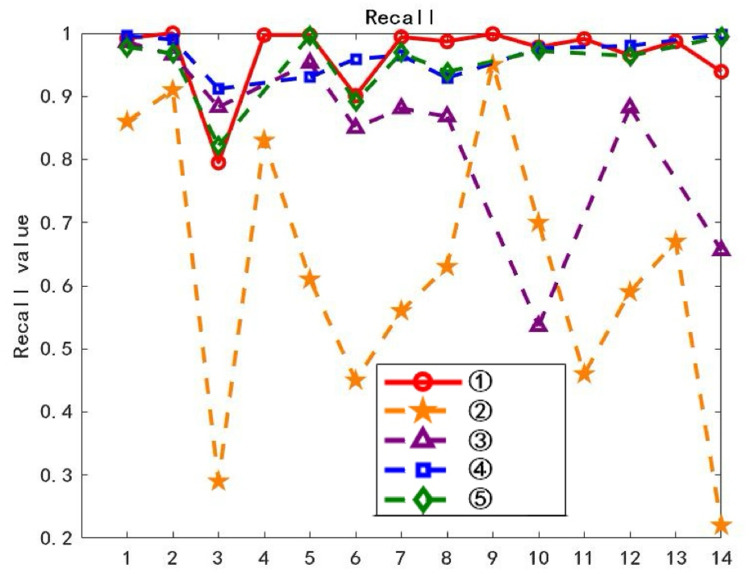
Comparison of Recall Metrics for the Five Algorithms.

**Fig 17:**
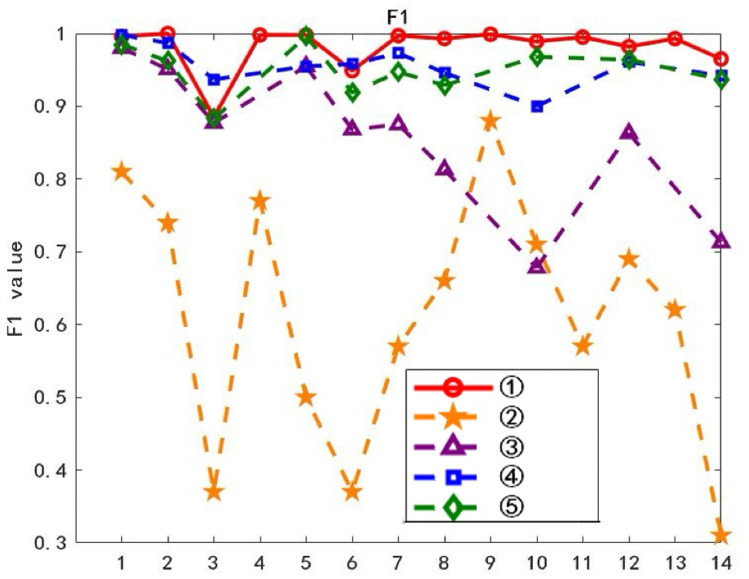
Comparison of F1 Score Metrics for the Five Algorithms.

(e) For a visual representation of the tables above, see Figs 15–17.
(f) The experimental results of the five algorithms on three different datasets are shown in Tables 15–17.

***Analysis of Calculation Results:***

① The experimental data obtained from Tables 12–14 are analyzed as follows:

In terms of Precision, the ATF-DF-WA algorithm performs almost perfectly across 14 text categories, with a Precision of 1.0 in 10 categories. The Precision for the remaining 4 categories is higher than 0.99. This indicates that the ATF-DF-WA algorithm achieves extremely high accuracy in classification tasks, with a very low error rate. Compared to the other four baseline algorithms, ATF-DF-WA shows a significant improvement in Precision, with an average increase of 2.80% to 80.36%. This improvement not only reflects the model’s enhanced ability to accurately identify samples but may also be related to the advantage of using wavelet-based feature selection in the ATF-DF-WA algorithm, which allows it to more effectively capture the feature differences between text categories, thereby optimizing classification performance.

In terms of Recall, the ATF-DF-WA algorithm has an average Recall of 0.965, outperforming the other four algorithms, particularly in entertainment text, where Recall reaches 1.0. This indicates that the ATF-DF-WA algorithm is able to identify more true positive samples, even in cases of class imbalance, effectively reducing the miss rate. Compared to other algorithms, ATF-DF-WA’s Recall improved by 0.10% to 54.65% on average. It is worth noting that, despite the overall excellent performance, the ATF-DF-WA algorithm performs slightly worse in classifying home-related texts compared to Algorithms ② and ③. This may be due to unclear category features or uneven data distribution in the home category. Future optimization work may need to further focus on this category, such as optimizing the selection of the optimal dimension or wavelet basis.

In terms of F1 score, the ATF-DF-WA algorithm achieved high F1 scores across all text categories, with an average of 0.981, outperforming the other four algorithms, particularly in entertainment texts, where the F1 score is 1.0. This suggests that the algorithm not only has high Precision but also effectively mitigates the negative impact of low Recall on overall performance. The F1 score, which combines Precision and Recall, provides a more comprehensive evaluation, indicating that the ATF-DF-WA algorithm is able to balance classification accuracy while capturing as much relevant information as possible in practical applications. Compared to other algorithms, ATF-DF-WA’s F1 score improved by 2.62% to 60.82% on average. The F1 score remained high across all categories, except for the home category, where it was slightly lower than Algorithm ④, further supporting the need for targeted optimization for this category.

② The experimental data obtained from Tables 15–17 are analyzed as follows:

The experimental results further validate the versatility of the ATF-DF-WA algorithm across multiple datasets. Whether in terms of Precision, Recall, or F1 score, ATF-DF-WA’s performance is significantly better than that of the other four algorithms across all three datasets. Specifically, Precision increased by 8.21% to 69.56%, Recall increased by 6.60% to 75.00%, and F1 score increased by 7.93% to 76.82%. These results demonstrate that the ATF-DF-WA algorithm not only performs outstandingly on a single dataset but also consistently delivers superior classification performance across different datasets, showcasing its excellent generalization ability and cross-task adaptability.

#### 4.3.4 Sub-experiment 3: text classification results and analysis of ATF-DF-WA vs. pre-trained model baseline algorithms.

In addition to traditional baseline algorithms, pre-trained models such as BERT have also demonstrated good performance on smaller datasets through fine-tuning. Therefore, this section further conducts a brief comparative study between ATF-DF-WA and such algorithms.

***Dataset and Baseline Algorithm Selection:*** Reference [38] investigated the text classification performance of pre-trained BERT models and proposed a method of fine-tuning the BERT model and embedding it into CNN and RNN deep learning models to improve the accuracy and stability of news text classification. The same THUCNews dataset was also used in this study, facilitating a comparison of results. Therefore, this paper selects the three baseline algorithms reported in reference [38], namely BERT, BERT-CNN, and BERT-RNN, for the comparative study.

***Experimental Calculation Results:*** The ATF-DF-WA algorithm is compared with the three baseline algorithms in terms of average Precision, average Recall, average F1 score, experimental running environment parameters, and training time. The results are shown in Table 18.

**Table 18:** Comparison of Text Classification Performance Between ATF-DF-WA and Pre-trained Model Baseline Algorithms.

Comparison Metrics	ATF-DF-WA	BERT-CNN	BERT-RNN	BERT
Precision(Average)	0.992	0.941	0.936	0.938
R(Average)	0.965	0.940	0.935	0.937
F1 Score (Average)	0.981	0.940	0.935	0.937
Operating Environment Parameters	CPU:AMD R7 6800H, Random Access Memory 16 GB	CPU:AMD R5 3600, GPU:NVIDIA GTX 1060(6 GB), Random Access Memory 16 GB	CPU:AMD R5 3600, GPU:NVIDIA GTX 1060(6 GB), Random Access Memory 16 GB	CPU:AMD R5 3600, GPU:NVIDIA GTX 1060(6 GB), Random Access Memory 16 GB
Training Time	1.98	3.50	/	/

Among these, the data for the first four evaluation metrics for the ATF-DF-WA algorithm comes from Table 18 of this paper, while the data for the three baseline algorithms comes from reference [38]. The data for the fifth evaluation metric (in hours) corresponds to the average value calculated from 20 independent training sessions for the ATF-DF-WA algorithm. The data for the BERT-CNN algorithm comes from the related study in reference [39], while the data for the BERT-RNN and BERT algorithms have not been reported in reference [39].

***Analysis of Experimental Results:*** The data obtained from Table 18 are analyzed as follows:

In the text classification task, the ATF-DF-WA algorithm outperforms the three baseline algorithms in all evaluated metrics. Compared to the best-performing baseline algorithm, BERT-CNN, the ATF-DF-WA algorithm shows a 5.42% improvement in average Precision, a 2.66% improvement in average Recall, and a 4.36% improvement in average F1 score. This indicates that the ATF-DF-WA algorithm not only shows a significant improvement in correctly classifying positive examples (Precision), but also captures more positive examples in terms of Recall, resulting in a more balanced and superior overall performance.

From the perspective of training time and hardware requirements, the ATF-DF-WA algorithm uses only an AMD R7 6800H CPU and 16GB of RAM for computation, whereas the BERT-CNN algorithm utilizes more powerful hardware, including an AMD R5 3600 CPU, an NVIDIA GTX 1060 (6GB) GPU, and 16GB of RAM, with GPU acceleration for computation. Despite using GPU acceleration, the training time for BERT-CNN is still 3.5 hours, whereas ATF-DF-WA completes its training in just 1.98 hours. Without GPU acceleration, ATF-DF-WA demonstrates high efficiency, finishing the training in a significantly shorter time. This reveals its substantial time advantage, especially in resource-limited environments.

In conclusion, the ATF-DF-WA algorithm outperforms pre-trained model baseline algorithms in terms of classification accuracy, recall capability, and computational efficiency. Moreover, it demonstrates superior training efficiency, particularly in environments with limited computational resources.

### 4.4 Experimental Conclusion of the ATF-DF-WA Algorithm

The ATF-DF-WA algorithm demonstrates significant advantages in text classification tasks. By combining the ATF-DF algorithm with wavelet analysis, this algorithm efficiently extracts text features and achieves high Precision, Recall, and F1 scores across different text categories. The key conclusions are as follows:

(1) Improvement Over Traditional Baseline Algorithms, Compared to traditional baseline algorithms, the ATF-DF-WA algorithm shows improvements in all evaluated metrics, especially excelling in handling complex text categories.
(2) When compared to pre-trained model baseline algorithms, ATF-DF-WA also demonstrates superior performance in terms of text classification metrics and training time, highlighting its strong application potential.

## 5 Conclusions

### 5.1 Main conclusions

This paper proposes a novel text category feature extraction algorithm, ATF-DF, and further introduces a new ATF-DF-WA text classification algorithm by integrating wavelet analysis theory. The main conclusions of this study are as follows:

(1) The ATF-DF algorithm has accurate text category feature extraction capabilities: The ATF-DF algorithm can effectively extract feature vectors for text categories. The experimental results indicate that: ① The feature terms in the feature vectors proposed by this algorithm are closely related to text categories, and their feature value weights accurately reflect the representativeness and distinguishing capability of these terms within the text categories. ② Compared to the TF-IDF algorithm, the ATF-DF algorithm improved Precision by 2.80% and 80.36%, Recall by 0.10% and 54.65%, and F1 score by 2.62% and 60.82% on two text classification baseline algorithms, respectively. This indicates that ATF-DF has an advantage over TF-IDF in text category feature extraction performance.
(2) The ATF-DF-WA algorithm has a performance advantage: Compared to the four traditional shallow learning and deep learning baseline algorithms, the ATF-DF-WA algorithm performs exceptionally well in text classification tasks. The experimental results indicate that: the average Precision increased by 2.80% to 80.36%, the average Recall increased by 0.10% to 54.65%, and the average F1 score increased by 2.62% to 60.82%, demonstrating that this algorithm has a significant advantage in text classification performance.Compared to baseline algorithms based on pre-trained models, it also demonstrates advantages in both classification performance and training speed.
(3) The ATF-DF-WA algorithm has application advantages: The ATF-DF-WA algorithm not only fully leverages the statistical advantages of large data samples but also overcomes the drawbacks of deep learning algorithms, such as high data and computational resource requirements, poor interpretability, and complex parameter tuning. Therefore, it is a lightweight solution suitable for environments with limited training data and constrained computational resources.
(4) Exploration of introducing wavelet analysis: This study effectively applies wavelet analysis theory and tools to the field of text classification, providing a valuable exploration for innovation in this area.

### 5.2 Limitations and potential constraints

Despite the achievements of this study, there are some potential limitations:

(1) Limitations in feature representation: The current text-to-waveform conversion process retains only term frequency features, ignoring the positional relationships and interactions between terms. This restricts the model’s representational capacity and its ability to capture fine-grained information.
(2) Insufficient multilingual support: The ATF-DF-WA algorithm has been evaluated solely on Chinese texts, and its applicability to other languages has not yet been assessed. Therefore, the algorithm may face performance degradation or adaptability challenges when handling texts in different languages.

### 5.3 Future outlook

Future research will focus on the following aspects: (1) Retaining more textual information in the typical feature vectors to construct feature vectors with richer information, which can then be mapped into high-dimensional waveforms or even images for further processing. (2) Further expanding on the ideas presented in this study by exploring the integration of additional tools from signal processing and image processing with deep learning frameworks, with the aim of generating more research outcomes through interdisciplinary convergence. (3) Combining the ATF-DF algorithm with deep learning models, such as using the extracted text category feature vectors as input for neural networks (e.g., LSTM or Transformer). This approach could further enhance the accuracy of text classification and fully leverage the powerful representational capabilities of deep learning models. (4) Combining the ATF-DF algorithm with deep embedding methods, for example, by integrating the feature vectors generated by ATF-DF with vectors produced by deep embedding at the feature level. This can be achieved through techniques such as vector concatenation or weighted averaging. Alternatively, during model training, different sources of feature vectors can be used as inputs to build a hybrid model. (5) Transforming the feature vectors generated by ATF-DF into a format suitable for input into large models for prompt-tuning, in order to further enhance the classification performance of these large models. (6) Developing more shallow learning and deep learning text classification algorithms based on the ATF-DF algorithm.(7) Optimize and adapt the ATF-DF algorithm to support text classification tasks in multilingual environments.(8) Introduce automated parameter adjustment mechanisms or develop new wavelet basis functions to accommodate a broader range of task scenarios, thereby further enhancing the generality and applicability of the method.
